# Mesenchymal stem cells-derived extracellular vesicles for therapeutics of renal tuberculosis

**DOI:** 10.1038/s41598-024-54992-z

**Published:** 2024-02-24

**Authors:** Natalia Yudintceva, Danila Bobkov, Maksim Sulatsky, Natalia Mikhailova, Elena Oganesyan, Tatiana Vinogradova, Alexandr Muraviov, Anna Remezova, Evdokia Bogdanova, Irina Garapach, Olga Maslak, Dilyara Esmedlyaeva, Marina Dyakova, Petr Yablonskiy, Rustam Ziganshin, Sergey Kovalchuk, Natalya Blum, Shirish H. Sonawane, Avinash Sonawane, Ankita Behl, Shailja Singh, Maxim Shevtsov

**Affiliations:** 1 Institute of Cytology of the Russian Academy of Sciences (RAS), Tikhoretsky Ave., 4, Saint Petersburg, Russia 194064; 2 Personalized Medicine Centre, Almazov National Medical Research Centre, Akkuratova Str. 2, Saint Petersburg, Russia 197341; 3 Saint-Petersburg State Research Institute of Phthisiopulmonology of the Ministry of Healthcare of the Russian Federation, Ligovsky Ave., 2-4, Saint Petersburg, Russia 191036; 4 Private University St. Petersburg Medico-Social Institute, Kondratievskiy Ave., 72A, Saint Petersburg, Russia 195271; 5 Shemyakin-Ovchinnikov Institute of Bioorganic Chemistry Russian Academy of Sciences, Miklukho-Maklaya Str., 16/10, Moscow, Russia 117997; 6 Kirov Military Medical Academy, Akademika Lebedeva Str., 6, Saint Petersburg, Russia 194044; 7 National Institute of Technology Warangal, Warangal, 506004 India; 8 Indian Institute of Technology Indore, Indore, 453552 India; 9 Special Centre for Molecular Medicine, Jawaharlal Nehru University, New Delhi, 110067 India; 10 Department of Radiation Oncology, Central Institute for Translational Cancer Research (TranslaTUM), Klinikum Rechts der Isar, Technical University of Munich, Munich, Germany; 11 School of Medicine and Life Sciences, Far Eastern Federal University, Campus 10 Ajax Bay, Russky Island, Vladivostok, Russia 690922

**Keywords:** Mesenchymal stem cells, Extracellular vesicles, Proteins, Renal tuberculosis, Anti-tuberculosis therapy, Microbiology, Diseases

## Abstract

Extrapulmonary tuberculosis with a renal involvement can be a manifestation of a disseminated infection that requires therapeutic intervention, particularly with a decrease in efficacy of conventional regimens. In the present study, we investigated the therapeutic potency of mesenchymal stem cell-derived extracellular vesicles (MSC-EVs) in the complex anti-tuberculosis treatment (ATT). A rabbit model of renal tuberculosis (rTB) was constructed by injecting of the standard strain *Mycobacterium tuberculosis* H37Rv into the cortical layer of the kidney parenchyma. Isolated rabbit MSC-EVs were intravenously administered once as an addition to standard ATT (isoniazid, pyrazinamide, and ethambutol). The therapeutic efficacy was assessed by analyzing changes of blood biochemical biomarkers and levels of anti- and pro-inflammatory cytokines as well as by renal computed tomography with subsequent histological and morphometric examination. The therapeutic effect of therapy with MSC-EVs was shown by ELISA method that confirmed a statistically significant increase of the anti-inflammatory and decrease of pro-inflammatory cytokines as compared to conventional treatment. In addition, there is a positive trend in increase of ALP level, animal weigh, and normalization of ADA activity that can indicate an improvement of kidney state. A significant reduction of the area of specific and interstitial inflammation indicated positive affect of MSC-EVs that suggests a shorter duration of ATT. The number of MSC-EVs proteins (as identified by mass-spectometry analysis) with anti-microbial, anti-inflammatory and immunoregulatory functions reduced the level of the inflammatory response and the severity of kidney damage (further proved by morphometric analysis). In conclusion, MSC-EVs can be a promising tool for the complex treatment of various infectious diseases, in particularly rTB.

## Introduction

Tuberculosis (TB) remains a global public health problem and is caused by a *Mycobacterium tuberculosis* (*Mtb*)^1^. The WHO guidelines published in 2022 include a strong recommendation for a six-month regimen of anti-tuberculosis drugs (ATDs) such as isoniazid, rifampicin, ethambutol, and pyrazinamide for people with drug-susceptible TB. However, prolonged and excessive use of ATDs often leads to antibiotic resistance, which has become a serious problem for lives and health of people around the world^2^. Treatment for people diagnosed with resistant and multidrug-resistant TB is more difficult and requires drugs that cause more side-effects. In addition, ATDs are potentially highly toxic to the liver and cause its drug-induced damage^3^. The development of TB largely depends on the reactivation of its dormant form against the background of the emerging immunodeficiency^4^. All these factors often prevent the implementation of complete and continuous anti-tuberculosis treatment (ATT) and are associated with a high risk of treatment failure^5^, development of acute liver failure, cirrhosis and death^6^. Thus, the development of new approaches to the treatment of TB is one of the main goals of translational medicine. For improvement of ATT efficiency, the methods modulated the body's immune defense can be used.

Currently, certain preclinical and clinical trials evaluate the potential of ATT especially based on the mesenchymal stem cells (MSCs) and MSC-derived extracellular vesicles (MSC-EVs)^7,8^.

It is known that the immunomodulatory and other therapeutic effects of MSCs are largely mediated by paracrine signals via extracellular vesicles (EVs)^9–11^. EVs secreted by MSCs have a number of advantages compared with stem cells. They have lower immunogenicity and a high safety profile^12,13^, exhibit excellent biotolerance^14^, and have unique targeting and delivery functions^15^. Thus, interactions between MSC‐EVs and immune cells make them an ideal therapeutic candidate for infectious, inflammatory, and autoimmune diseases^16^.

EVs constitute a heterogeneous population of nanoparticles encapsulated in a lipid membrane carrying various biomolecules such as RNA (mRNA and microRNA) and proteins (e.g., membrane receptors, enzymes, cytokines, and growth factors). MSC-EVs contain active molecules that support immune response (e.g., interleukin-6 (IL-6), IL-10, indoleamine 2,3-dioxygenase (IDO), prostaglandin E2 (PGE), etc.), have anti-inflammatory (IL-4, IL-13, tumor necrosis factor-α (TNF-α)), antiviral/antibacterial (lysozyme, cystanins, etc.) effects^17–19^ and promote epithelial repair and tissue regeneration (transforming growth factor (TGF), keratinocytes growth factor (KGF), vascular endothelial growth factor (VEGF),hepatocyte growth factor (HGF), etc.)^20^. MSC-EVs exert immunomodulatory effect on various immune system cells by the regulation of the Th1/Th2 balance^21^, stimulation of regulatory T cells (Treg and Th2)^22,23^, inhibition of B cells activity^24,25^. Furthermore, MSC-EVs were shown to have anti-inflammatory activity by decreasing TNF-α and increasing TGF-β, IL-10^26,27^.

One of the most common extrapulmonary form of tuberculosis is genitourinary (renal) tuberculosis (rTB) with the severe pathomorphological changes of kidneys. The formation of granulomas, their calcification, as well as the processes of specific inflammation lead to the gradual loss of renal function and progression of fibrosis. Earlier we demonstrated that intravenous administration of MSCs results in accumulation and retention of injected cells in the renal parenchyma in the model of rTB^28^. Previously, in several preclinical studies it was shown that EVs promote tissue repair, reduce inflammation in different models of acute kidney injury^29^ and are effective in the reversion of renal fibrosis in a diabetic nephropathy model^30^. EVs accelerated repair of damaged tubular cells by promoting cell proliferation and protection of cells from apoptosis^31^. The transfer of miRNAs from MSC-EVs to target cells has been demonstrated as the main mechanism for reducing kidney damage^32^.

In the clinical practice, to assess the state of the rTB kidneys and the severity of the inflammatory reaction some biochemical blood biomarkers such as creatinine (CR), alkaline phosphatase (ALP), albumin (AL), ceruloplasmin (CP), marker of purine metabolism (adenosine deaminase activity (ADA) and marker of destruction and remodeling—elastase (EL) are analyzed. In addition, TB progression or regression may be indicated by changes of the anti -or pro-inflammatory cytokines levels. In the present work, we evaluated the therapeutic efficacy of MSC-EVs in combination with a standard anti-TB treatment in the preclinical model of rabbit rTB using biochemical, histological and morphometry methods, and computed tomography (CT).

## Results

### Evaluation of the MSC-EVs

**Figure 1:**
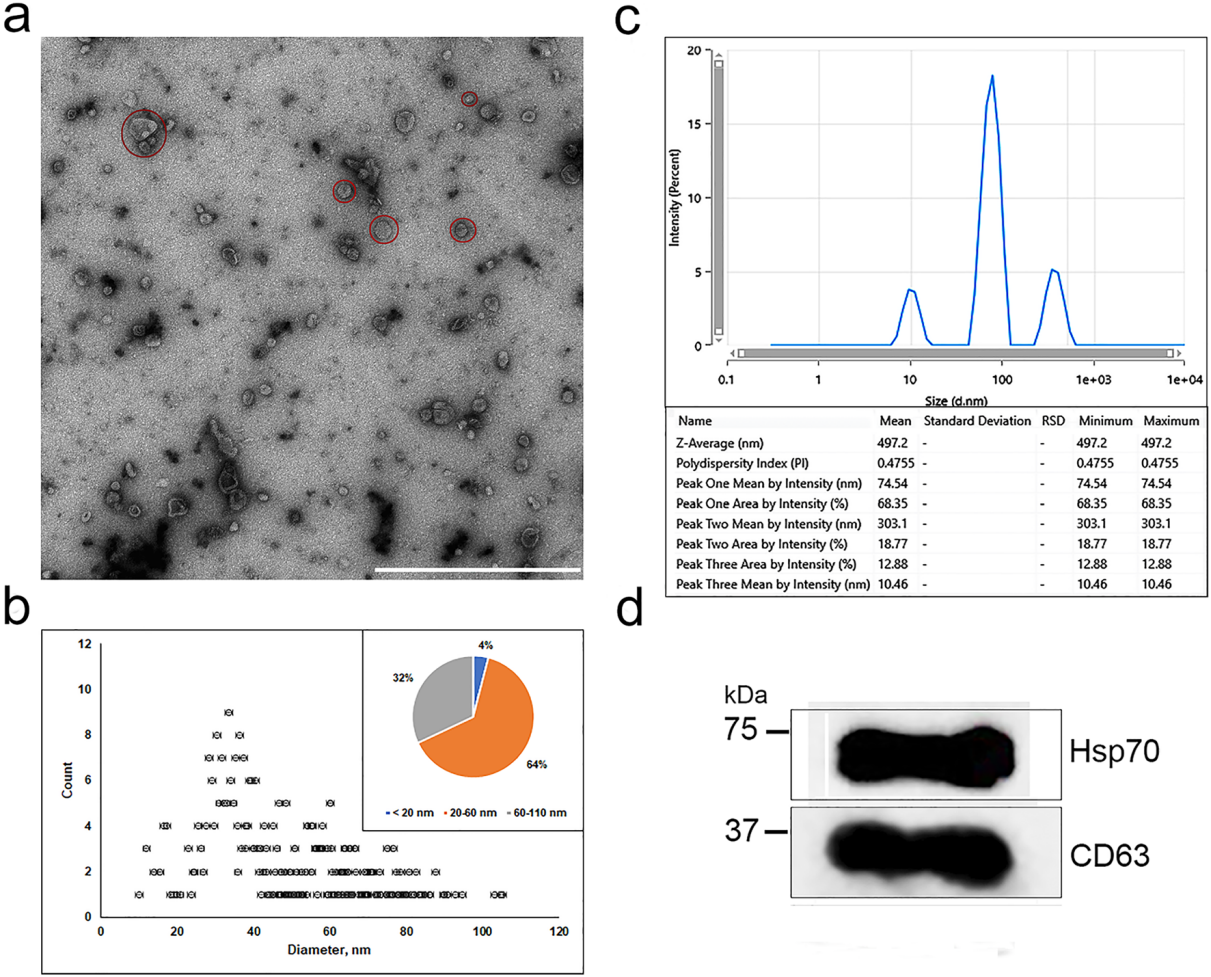
Characterization of MSC-EVs. (**a**) TEM of MSC-EVs. Red circles indicate single EVs and its group. (**b**) Size distribution of MSC-EVs measured by TEM, detecting EVs from 20 to 110 nm. Percentage of MSC-EVs present within the size ranges of ≤ 20, 20–60, and 60–110 nm. (**c**) The hydrodynamic size distribution of MSC-EVs measured by DLS. Percentage of MSC-EVs present within the size ranges of 10 ± 0.5 nm; 70 ± 5.1 nm, and 300 ± 3.3 nm. (**d**) Western blot analysis of MSC-EVs showed enrichment of known EV markers Hsp70 and CD63.

Isolated MSC-EVs were characterized by morphology, size, specific markers, and protein content. We confirmed the MSC-EVs exhibited the spherical morphology using TEM (Fig. 1a). Analysis of our results showed that the diameter of MSC-EVs reached 105 ± 5 nm (while 70% of the observed vesicles were up to 60 ± 3 nm in diameter) (Fig. 1b). The data obtained were in good agreement with one as determined by Zetasizer Nano. According to the dynamic light scattering of MSC-EVs solution, the three groups of EVs had a diameter range of 10–300 nm (10 ± 0.5 nm; 70 ± 5.1 nm, 300 ± 3.3 nm) (Fig. 1c). Probably, the presence of a certain number of vesicles with large sizes can be explained by the presence of aggregation during measurement. Western blot analysis showed that the MSC-EVs were positive for conventional EV-specific markers including Hsp70, CD63 (Fig. 1d, Supplementary Fig. S1). Total protein concentration in MSC-EVs probes (n = 3) was equal to 200 µg/mL.

### Characterization of MSC-EV proteome by mass spectrometry

**Figure 2:**
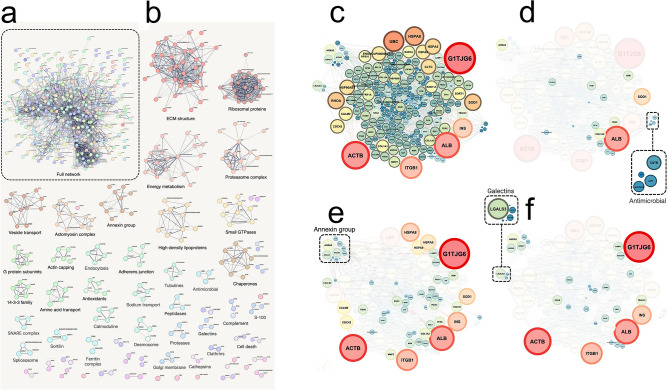
PPI analysis of MSC-EV proteome. (**a**) Full STRING PPI network. (**b**) STRING PPI network was divided into clusters by the MCL algorithm. For each cluster with more than two proteins, assigned name represents one of three subontologies (biological process, protein complex, or functional group). Cluster sizes are represented by different colors. (**c**) PPI network of MSC-EV proteome extracted from STRING database with node sizes rescaled according to node degree. Some protein clusters were isolated from this network to identify cluster interactors, and only proteins that interact with the proteins of the cluster according to the STRING database were marked: antimicrobial (**d**), Annexin group (**e**), and Galectins (**f**).

To investigate the proteomic profile of isolated MSC-EVs, we performed quantitative liquid chromatography-tandem mass spectrometry (LC–MS/MS) proteomics analyzes on these EVs. The total of 474 proteins were identified including EV biomarkers such as heat shock proteins, Annexins, and CD63 (Fig. 2a, Supplementary Table S1). MCL clustering reveal 32 groups with more than two proteins, among them three largest were extracellular matrix (ECM) proteins, proteasome complex, and energy metabolism group (Fig. 2b). Among MSC-EVs proteome were found proteins known to be associated with antimicrobial and anti-inflammatory activity, and proteins involved in immunity (Fig. 2, Supplementary Tables S2–S3). Some of these proteins are listed in Table 1.

**Table 1:** List of featured EVs proteins revealed by mass spectrometric analysis.

Protein name	Cluster name	Biological activity	References
Hemoglobin subunit beta-2 Lactotransferrin Lysozyme Cystatin B	Antimicrobial	Immune response Antimicrobial defense Regulation of inflammation cytoskeleton modulation	^48–50^
Annexin A	Annexin group	Anti-inflammatory response	^53‒56^
Apolipoprotein E Apolipoprotein A-I	High-density lipoproteins	Lipid binding	^57‒59^
Galectin Galectin-3	Galectins	Cell adhesion Inflammatory response	^51,52^

### Assessment of rTB progression

**Figure 3:**
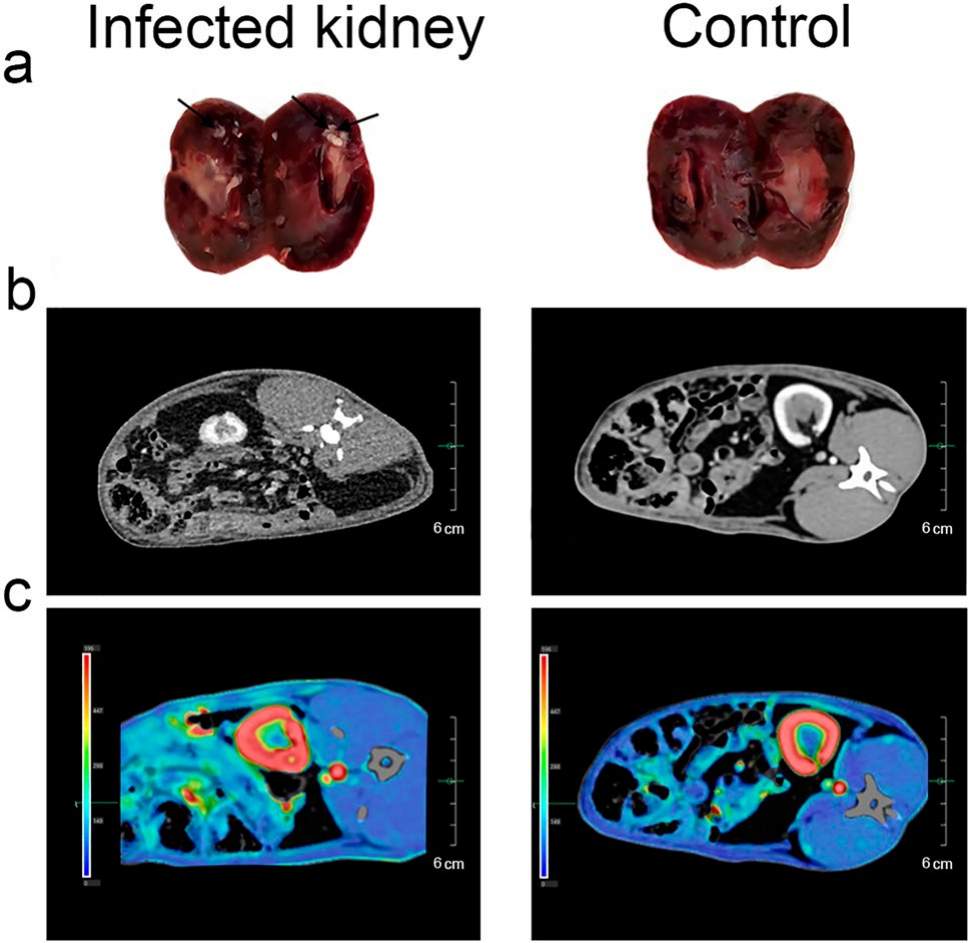
Assessment of the development of rTB. (**a**) Macrophotos of the cross section of the kidney. Black arrows show foci of TB development. Abdominal CT: (**b**) the zone of inflammation is seen as the hypovascular area in the cortical and medullar layer on the left and intact kidney on the right. (**c**) CT perfusion of the kidney: hypoperfusion region in the kidney that correlates with area of destruction (green colour) on the left and normal layer differentiation with no destruction areas on the right.

The assessment of the rTB progression was performed in 18 days after infection of *Mtb* by visual assessment of the kidneys, CT scan and assessment of the most significant biochemical blood parameters. The pathological changes of the kidney were detected as the foci of *Mtb*. As a control was used healthy rabbit (Fig. 3a). In addition, computed tomography (CT) was performed. Within 30 s, sequential images of the abdomen were obtained and served as the basis for the perfusion map. CT of the abdominal cavity revealed a clearly defined zone of reduced perfusion in the left (infected) kidney and a focus of destruction (Fig. 3b,c).

#### Assessment of the blood biochemical indicators

In addition, by the 18th day after infection various blood biomarkers were evaluated. Compared to the baseline values, for all rabbits statistically significant decrease of AL level (from 0.015 to 0.013* g/L), ALP activity (from 178.0 to 123.0** U/L), and an increase of ADA activity (from 13.4 to 21.2** U/L) and rabbits weight (from 3148.0 to 3454.0** g) were showed. The levels of CR, CP and EL had no statistically significant changes (Table 2).

**Table 2:** Biochemical indicators of the blood.

Indicators	Baseline values	Examination groups, 18 days	Examination groups, three months		
			1	2	3
CR μmol/L per g. weight	0.025	0.021	0.023	0.016*	0.02**
ALP U/L	178.0	123.0**	41.0*	41.0*	51.0**
Al g/L per g. weight	0.015	0.013*	0.013*	0.01**	0.01**
CP g/L per g. weight	0.0001	0.0001	0.0001	0.00006*	0.00008**
Total ADA U/L	11.8	21.2**	11.1*	14.3*	13.6**
EL IU	221.0	304.3	412.9**	376.7*	449.2*
Weigh, g	3148.0	3454.0**	3628.0*	3956.0**	4240.0*

Examination groups: (1) challenge control (without ATT); (2) standard ATT; (3) standard ATT + MSC-EVs.

*CR* creatinine, *ALP* alkaline phosphatase, *AL* albumin, *CP* ceruloplasmin, *Total ADA* adenosine deaminase activity, *EL* elastase, *Asterisks* statistically significant difference shown for the groups when compared to the baseline, p-values are expressed as follows: *p ≤ 0.05; **p ≤ 0.01.

In three months after start of the experiment, for assessment of rTB progression, an ELISA, histological and morphometric studies were added. However, the progression of rTB was detected mainly for the first group. Significant changes of the same blood parameters compared with the baseline for all groups were assessed using biochemical method. Thus, ALP activity had further decreased (from 178.0 to 41.0* U/L) and to a lesser extent for the third group (to 51.0* U/L) and EL level had further increased compared to the baseline. Animals of the first group had minimum weigh (3628.0* g) and a maximum value of ADA (16.2* U/L) compared to the baseline and to other groups. Level of EL was increased and the levels of AL, CR, and CP had no significant changes (Table 2).

The minimum levels of anti-inflammatory cytokines (IL-4 and IL-10) throughout the entire observation period (from 1 day to 4 weeks after introduction of MSC-EVs) (from 16.77 to 10.4 pg/mL and from 7.15 to 6.41 pg/mL, respectively) were revealed for the first group. At the same time the levels of pro-inflammatory cytokines (IFN-γ and TNF-α) for this group had maximum values throughout the entire observation period (from 53.06 to 48.26 pg/mL and from 29.19 to 22.71 pg/mL, respectively). The most significant differences between groups were identified after a two-week follow-up. Thus, IFN-γ level for the first group was 71.1 pg/mL while for the second and third groups these values were 55.27 and 54.51 pg/mL, respectively (Table 3).

**Table 3:** Concentration of cytokines in blood plasma.

Cytokines (pg/mL)												
	1				2				3			
	1st day	1st week	2nd week	4th week	1st day	1st week	2nd week	4th week	1st day	1st week	2nd week	4th week
IL-4	16.77	13.3	12.1	10.4	17.95	15.61	14.87*	14.07	18.36*	16.87	15.32	14.09*
IL-10	7.15	7.80	8.58	6.41	17.02**	18.45	18.36*	50.36*	50.66	40.77	47.11**	78.23*
IFN-γ	53.06	57.81	71.1	48.26	30.66	53.29*	55.27	36.53*	30.12	37.31	54.51	31.2**
TNF-α	29.19	29.43	22.57	22.71	18.58	15.9	15.41**	15.45*	18.02	15.99*	5.95*	5.78*

Examination groups: (1) challenge control (without ATT); (2) standard ATT; (3) standard ATT + MSC-EVs.

*CR* creatinine, *IL* interleukins, *IFN-γ* interferon γ, *TNF-α* tumor necrosis factor α, *Asterisks* statistically significant difference shown for the groups when compared to the first group, p-values are expressed as follows: *p ≤ 0.05; **p ≤ 0.01.

#### Histological and morphometric studies

**Figure 4:**
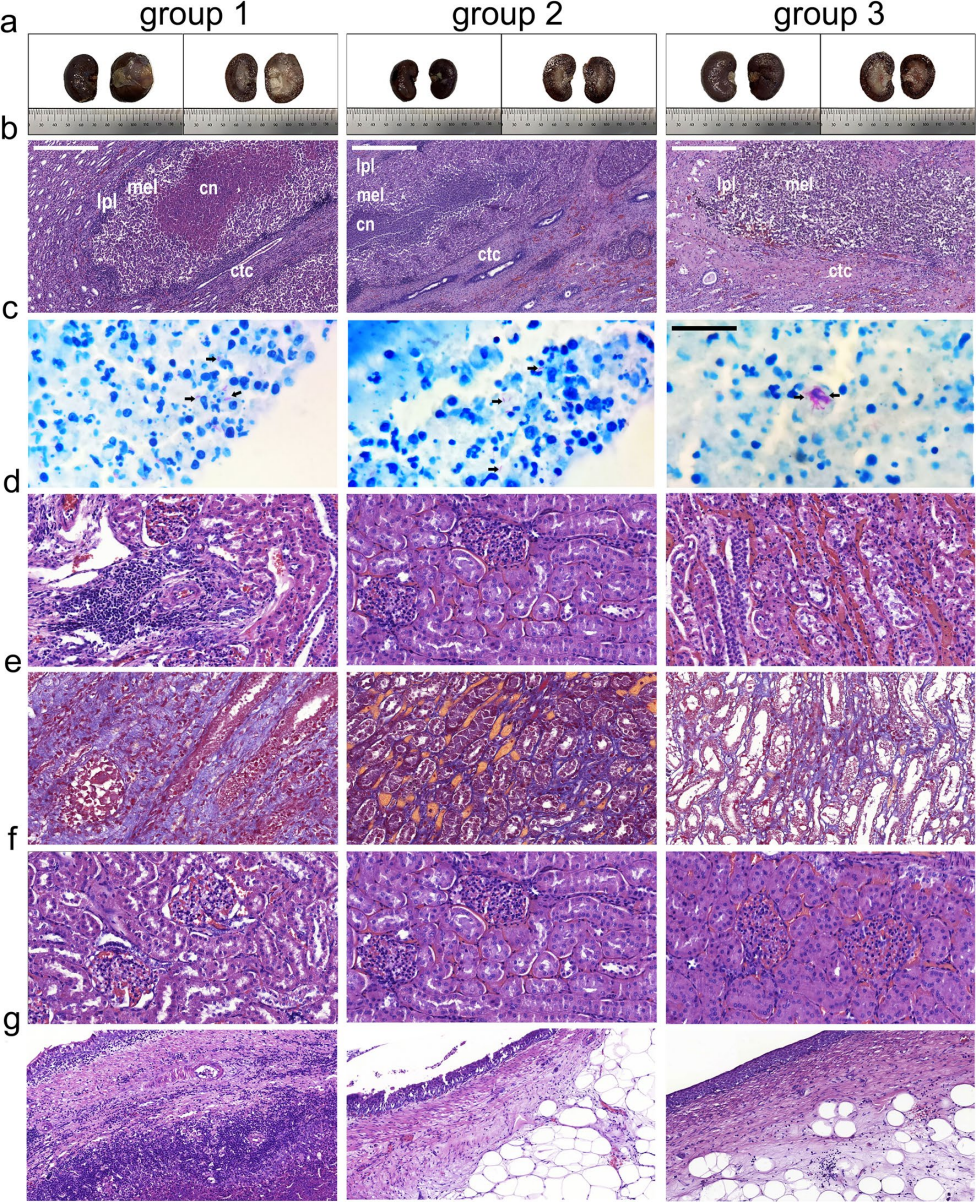
Histological section of the kidney. (**a**) Macrophotos of the kidney. Whole organ (left column); cross section (right column). Infected kidney (left), control kidney (right). (**b**) Active specific inflammation of kidney: caseous necrosis (cn); macrophage epithelioid layer (mel); lymphocyte-plasmacyte layer (lpl); connective tissue capsule (ctc). Stained with hematoxylin and eosin (G&E). Scale bar represents 100 μm. (**c**) Ziehl–Neelsen staining, black arrows show μTB. (**d**) Specific and interstitial inflammation of the cortex. (**e**) Distribution of collagen in the medulla, Masson's trichrome stain; (**f**) Kidney glomeruli. Scale bars represent 300 μm. (**g**) Renal pelvis. Scale bar represents 100 μm. (**d, f** and **g**) stained with G&E. Scale bar represents 300 μm (**c**–**g**).

The visual assessment of animals from first group showed bigger size of the left kidney than the right kidney. Histological analysis revealed that the cortical and medulla were differentiated and the pelvis was dilated (Fig. 4a). Caseous necrotic area (about 40.6 mm^2^) was mainly identified in the medulla. A connective tissue capsule at the border of caseous-necrotic masses was formed (Fig. 4b). Active specific inflammation with macrophage-lymphocytic infiltration and acid‐fast rods in the medulla (Fig. 4b,c, respectively) were observed only in the left but not the right kidney.

Data of morphometric study of left kidneys showed the area of specific inflammation had maximum value (40.6 ± 3.5 mm^2^) compared with other groups (6.5 ± 9.5 mm^2^ and 0.34 ± 0.04 mm^2^, respectively) (Fig. 4d and Table 4). One of the most of the quantitative indicators of kidney such as glomerular capillary diameter for animals of second and third groups (5.4 ± 1.1 and 5.5 ± 1.3 μm) compared with the first group (9.8 ± 0.9 µm) can be a sign of congestion in the infected kidney (Fig. 4f and Supplementary Table S4). Quantitative parameters of the renal vasculature were not significantly changed in both groups of treated and untreated animals with the exception of the wall thickness of the interlobular vein and its diameter that was smaller for first group as compared with other groups (Supplementary Table S6).

**Table 4:** Quantitative indicators of renal functional and structural changes.

Indicators	Examination groups			*p*
	1 (n = 5)	2 (n = 5)	3 (n = 5)	
Cortical width, mm	4.1 ± 0.42	3.5 ± 0.37	3.3 ± 0.31	0.30
Medulla width, mm	9.3 ± 0.8	12.1 ± 2.1	11.8 ± 1.3	0.48
Specific inflammation area, mm^2^	40.6 ± 3.5	6.5 ± 9.5	0.34 ± 0.04	0.048
Intestinal inflammation in cortical, %	7.1 ± 0.51	1.7 ± 1.6	2.0 ± 2.5	0.78
Intestinal inflammation in medulla, %	5.5 ± 1.5	5.7 ± 2.4	5.1 ± 2.8	0.73
Collagen area in cortical, %	6.7 ± 0.76	5.0 ± 4.1	6.4 ± 3.6	0.51
Collagen area in medulla, %	27.8 ± 2.5	10.1 ± 11.8	19.9 ± 7.6	0.15

*p* significance value for the comparison of second and third groups with the first group.

#### Computed tomography (CT) scan

**Figure 5:**
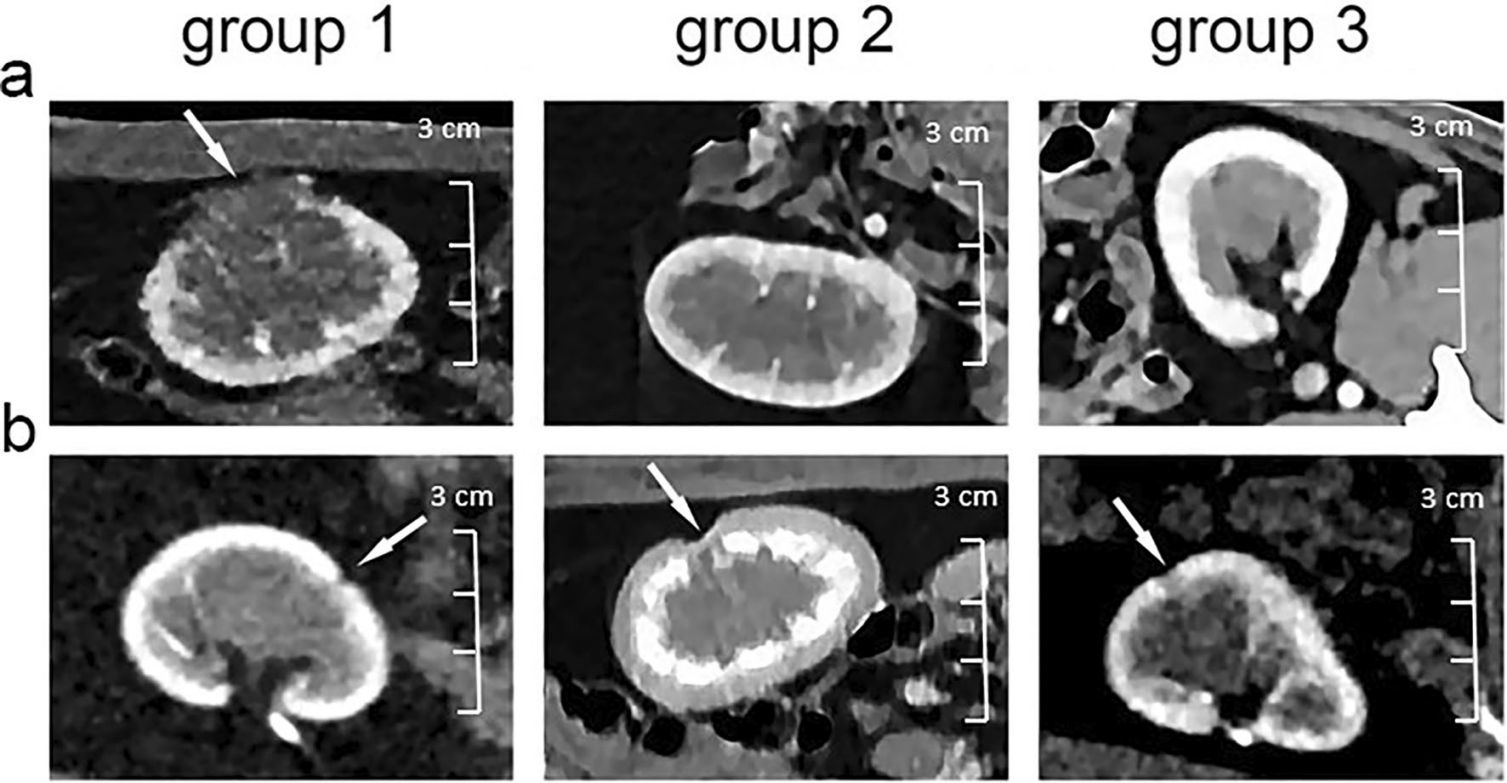
Computed tomography analysis, cortico-medullary phase (**a**) There is a massive hypovascular infiltration of the kidney in the first group and no changes in the second and third groups. (**b**) The slight retraction of the wall due to scarring in all animals of the first group and in one animal from second and third group. White arrows show on pathological changes of kidney.

The CT of the kidneys with injection of contrast agent for better visualization of the parenchyma changes was performed. The first group had the infiltration of the kidney (Fig. 5a) with massive hypovascular zone in cortical and medullar layers, and involvement of adjacent fat. The retraction of the wall due to scarring (Fig. 5b), thinning of the cortical layer, and fibrous post-inflammatory changes were observed.

### Assessment of the therapeutic effect of MSC-EVs

To assess the therapeutic efficiency of MSC-EVs, we compared biochemical markers of blood and plasma, data of histological and morphometric analysis and CT obtained for animals of the second and third groups in three months after ATT.

#### Assessment of the biochemical indicators

Some biochemical markers of blood such as ALP activity had further decreased compared with the baseline. However, ALP level was decreased to a lesser extent for the third group (from 178.0 to 51.0* U/L) compared with the second (41.0** U/L). Such important biochemical indicator as a total ADA had statistically significant value (13.6** U/L) similar to the baseline (13.4 U/L) and was less than in second group (14.3* U/L) (Table 1). EL level and animal weight for third group were higher than the second group (449.2* IU and 376.7* IU; 4240.0* g and 3956.0** g, respectively), and the CP level was lower (0.00008** g/L and 0.00006*, respectively) (Table 2). There were no differences of AL level between groups. The CR level had statistically significant differences between the groups. However, no significant increase was observed, which can indicate the absence of significant renal failure.

The levels of anti-inflammatory (IL-4 and IL-10) on the first day after MSC-EVs administration for the third group had the highest values (18.36* and 50.66 pg/mL, respectively) compared with the second group (17.86 pg/mL, 17.02** pg/mL, respectively). Especially the values of IL-10 level throughout the entire observation period for third group was many-fold higher as compared to the second group (Table 3).

On the first day pro-inflammatory cytokine IFN-γ level for second and third groups (30.66 and 30.12 pg/mL) had no difference and was significantly lower as compared to the first group (53.06 pg/mL). IFN-γ and TNF-α level had significantly decrease in 4 weeks for the second and third groups. However, for the third group IFN-γ and TNF-α levels were significantly lower compared to the second group (31.2** and 36.53* pg/mL; 5.78* and 15.45* pg/mL, respectively) (Table 3).

#### Histological and morphometric study

In three months, the animals of the second and third groups had not demonstrated difference between size of the left and right kidney (Fig. 4a). Cortical and medulla were differentiated without macroscopic signs of structural disturbance, the pelvis was not dilated. No specific TB inflammations or acid‐fast rods were found, excepted for one of the animals from each group (Fig. 4b,c, respectively). Data of morphometric study of left kidneys showed the smaller values of the area of specific and interstitial inflammation of the cortical layer and collagen area of the medulla in animals of the second and third groups compared with the first group. It should be noted that the area of specific inflammation in the third group (0.34 ± 0.04 mm^2^) was more than one hundred and twenty times smaller as compared with the first (40.6 ± 3.5 mm^2^) and second (6.5 ± 9.5 mm^2^) groups, respectively (Fig. 4d and Table 4).

Most of the quantitative indicators of kidney glomeruli between animals of second and third groups did not show any significant differences. However, some parameters indicated the lesser degree of remodeling and infiltration of the renal pelvis wall, in particular for the third group. Thus glomerular capillary diameter (5.4 ± 1.1 and 5.5 ± 1.3 μm, respectively) (Fig. 4f and Supplementary Table S4), the epithelium height (54.5 ± 26.8 and 66.5 ± 20.1 µm, respectively) (Fig. 4e and Supplementary Table S5) and the thickness of the renal pelvis (289 ± 198 and 377 ± 168 µm, respectively) (Fig. 4f and Supplementary Table S6) had smaller values compared with those for the second group. Quantitative parameters of the renal vasculature were not significantly changed in both group treated and untreated animals with the exception of the thickness wall of the interlobular vein and its diameter that was smaller for first group as compared with other groups (Supplementary Table S6).

#### Computed tomography (CT) scan

The destruction or infiltration in the parenchyma and zones of altered perfusion were not seen in the second and third groups by the CT of the kidneys (Fig. 5a). For only one animal from second and third groups, a slight retraction of the kidney was observed (Fig. 5b).

## Discussion

Currently, various studies are focused on the potential of stem cells for as an adjuvant therapy of TB. It has been suggested that cell therapy can limit tissue damage and transform unproductive inflammation into effective immune responses directed at the pathogen. MSCs are widely used in various fields of regenerative medicine^20,33^ and infectious diseases^34^. Furthermore, there has been considerable interest in the application of MSC-EVs instead of cells for the treatment of various diseases^11^. Compared with whole-cell therapy, MSC-derived EVs have many advantages such as good tolerability, low immunogenicity, no risk of malignancy and more stable membrane structure than MSCs etc. These advantages provide broader prospects for the treatment of diseases using cell-free therapy. However, the immunomodulatory effects of MSCs and MSC-EVs in *Mtb* infection remain largely unknown. In present study, we employed a rabbit model of rTB to analyze therapeutic effect of MSC-EVs protein content in combination with a standard ATBT.

Firstly, we have evaluated the progression of rTB. The progression of rTB was demonstrated by CT scan in 18 days after infection of *Mtb* (Fig. 3). In addition, changes of biochemical indicators over the entire observation period (in 18 days after infection and in three months after ATT) were compared with baseline values. Our data in 18 days and three months showed the development of the inflammatory process, renal failure and kidney damage for all experimental animals. In three months, we also observed further development of this process mainly for the first group, as evidenced by a further decrease of ALP level and an increase of a total ADA (Table 2). ALP can be released in soluble forms and is highly expressed by the intestine and kidneys, where it is considered a specific marker^35^. A significant decrease of ALP level has been shown using models of renal ischemic stroke^36,37^ and indicate the kidney damage^38^. The development of renal failure was also accompanied by an increase of ADA activity that is the enzyme of purine metabolism. These changes were associated with a decrease of extracellular adenosine levels, which reduces the glomerular filtration rate by constricting the afferent arterioles, especially in the superficial nephrons, and thus reduces the salt load and transport work of the kidneys in accordance with the concept of metabolic control of organ function^39^.

In addition, the rTB progression continued for the first group in three months that was shown by analyzing of cytokine level, histological and morphometric studies, and CT scan. The minimum level of anti-inflammatory (IL-4 and IL-10) and the maximum level of pro-inflammatory (INF-γ and TNF-α) cytokines (Table 3) indicated the inflammatory process and the presence of active TB form. Although the immunological mechanisms of TB are not yet fully understood, certain studies have suggested that the IFN-γ/TNF-a producing T cells are associated with active TB infection^40^. Data of histological and morphometric study, and CT scan also showed the big area of specific inflammation (Fig. 4, Table 4) and pathological changes in the kidneys (Fig. 5).

In the present study, therapeutic efficiency of MSC-EVs have been predominantly demonstrated by assessing changes of biochemical indicators and data of histological and morphometric analysis for the third group compared with the second group. Thus, there was a positive trend of increasing ALP level, animal weigh, and normalizing of ADA to the baseline value that can indicate an improvement kidney state (Table 2). ADA activity can be associated with adenosine-associated activation of A2A, A2B receptors. Signal transduction through A2A, A2B receptors reduces infiltration of T effector cells and M1 macrophages and promotes the formation of regulatory T cells and M2 macrophages associated with reduced inflammation and less renal fibrosis^41^. Change of EL level also was detected for the third group. However, EL is a regulator of the inflammatory response and in different situations can act as both a pro-inflammatory and anti-inflammatory agent^42^. One of the most indicator of kidney state CR had not significant change of its level, indicating the absence of significant renal failure.

In the present work, we found that the administration of MSC-EVs probably can induce the production of anti-inflammatory cytokines (IL-4 and IL-10) and reduce the level of pro-inflammatory cytokines (i.e., IFN-γ, TNF-α) (Table 3). ELISA methods confirmed a statistical increase in the level of anti-inflammatory cytokines compared with the conventional treatment (Table 3). The anti-inflammatory effect of MSC-EVs is based on the delivery of immunomodulatory proteins to inflammatory immune cells (dendritic cells (DCs), M1 macrophages, CD4 + Th1 and Th17 cells), which allows their phenotypic conversion into tolerogenic DCs, immunosuppressive M2 macrophages, and tolerogenic DCs and T-regulatory cells. In addition, MSC-EVs can activate autophagy and/or inhibit apoptosis, necrosis and oxidative stress in damaged cells, especially kidney cells, promoting their survival and regeneration.

Presumbaly, the injection of MSC-EVs had impact for immunity cells and increase production of IL-4 by T helper 2 (Th2), basophils and mast cells that perform critical functions in the immune response^43–45^. T cell immunity, mediated by such cytokines as IFN-γ and TNF-α, plays a key role in the control of TB infection^46^. IL-4 in T cells can induce differentiation of naive CD4 T cells into Th2 cells, in B cells it induces immunoglobulin (Ig) class switching to IgG1 and IgE, and in MPs it induces their alternative activation^44^. IL-10 is another the most important cytokine with anti-inflammatory properties, that has a central role in the pathogenesis of *Mtb* infection by limiting the immune response to pathogens and thereby preventing damage to the host^47,48^. IL-10 is synthesized by Th2 and MPs and stimulates the proliferation and differentiation of B-lymphocytes, suppresses the synthesis of IL-2 and IFN-γ by T-helper type-l (Thl) cells, and inhibits the production of pro-inflammatory cytokines. However, the action of IL-10 can have both stimulating and inhibitory effects on various types of immune reactions depending on which cells (helper and/or regulatory T-cells) secrete this cytokine^49^.

Morphometric analysis revealed significant differences between second and third groups in such quantitative indicators as an area of specific and interstitial inflammation in the medulla (Table 4), glomerular capillary diameter (Supplementary Table S4), the epithelium height (Supplementary Table S5) and the thickness of the renal pelvis (Supplementary Table S6), indicating the therapeutic effect of MSC-EVs.

The therapeutic effect of MSC-EVs can be associated with proteins that have antimicrobial, anti-inflammatory and immunomodulatory activity. A number of studies have shown that the therapeutic potential of MSCs is associated with the anti-bacterial activity of cells directed against various pathogens in particular *Escherichia coli, Pseudomonas aeruginosa, Staphylococcus aureus* through the secretion of anti-microbial peptides (AMPs)^17–19^. Probably the anti-microbial effectiveness can be associated with the capacity of AMPs to kill or slow down bacterial growth. However, it is not yet known whether MSC-EVs affect *Mtb* growth in the same way. Further studies on the effects of AMPs on different *Mtb* strains are required.

In the present work, we detected some AMPs (LTF, LZ and Cystatin B) (Table 1), which has numerous biological roles, including the modulation of immune responses, and has anti-microbial, anti-viral, anti-oxidant, anti-cancer, and anti-inflammatory activities^50^.

We detected proteins with anti-inflammatory activity (ANXA1 and APOE). ANXA1 appears to be one of the annexins most actively involved in anti-inflammatory responses using a variety of experimental models^51^.

The immunoregulatory function of MSC-EVs has been studied in many different works. It was shown MSCs involved in innate and adaptive immunity and their immunomodulatory functions are manifested mainly when interacting with immune cells (T-cells, B-cells, natural killer (NK) cells, macrophages (MPs), monocytes, dendritic cells, and neutrophils) through the formation of intercellular contacts and implementation of paracrine activity^52,53^. By influencing the adaptive immune system, T-cells in particular, MSCs inhibit the differentiation of Th17, inducing the production of IL-10 and PGE2, as well as inhibiting IL-17, IL-22, and IFN-γ^54^.

Our study did not investigate the therapeutic efficacy of individual vesicle proteins, which is a limitation of this study. However, we showed a significant difference between the standard therapy regimen and the combination of anti-TB drugs with MSC-derived vesicles. Significant decrease of an area of specific inflammation, normalization of biochemical markers, increase of the anti-inflammatory and decrease of pro-inflammatory level of cytokines can indicate immunomodulatory properties of MSC-EVs and consider them as a promising tool in the complex therapy of TB.

## Conclusions

We did not evaluate the therapeutic efficacy of individual specific proteins with different functions, which is a limitation of the study. However, it should be noted that the MSC-EVs effect may be due to the cumulative effect of the proteins they contain. To enhance the therapeutic effect of cell-free therapy, various approaches are possible, in particular, an increase in the concentration of total protein, the frequency of administration, as well as an increase in the content of proteins with antibacterial and immunomodulatory effects in extracellular vesicles. In addition, EVs contain not only proteins, but also lipids and nucleic acids, growth factors, etc., and their participation in the complex action can also be assumed. One of major challenges in developing cell-free therapies is a large variation in therapeutic efficacies of MSC-EVs depending on differences in donors and tissue sources, cell culture conditions, frequency of EV administration and stage of the diseases and other. Besides it is very important the standardization of EVs isolation, storage, and characterization to ensure universality and reproducibility of results. Presumably, large-scale manufacturing methods for the clinical application of EVs could be developed to improve therapeutic efficacy of the treatment of infectious diseases, in particular TB.

## Methods

### Ethics statement

Twenty male Chinchilla rabbits (weight 3500 ± 250 g) were obtained from FSUE Nursery of laboratory animals “Rappolovo” of the Federal State Budgetary Institution National Centre Kurchatov Institute (St.Petersburg, Russia). All animal studies had been approved by the Independent Ethics Committee of the Saint‐Petersburg State Research Institute of Phthisiopulmonology of the Ministry of Health of the Russian Federation and were in accordance with GOST 33216‐2014 “Rules for working with laboratory rodents and rabbits” and in accordance with ARRIVE guidelines (https://arriveguidelines.org).

### MSC-EVs isolation

Rabbit MSCs were isolated from bone marrow as previously described^55^ and corresponded to multipotent mesenchymal stromal cells based on the Minimal criteria of The International Society for Cellular Therapy^56^. The cells were cultured in DMEM/F12 medium containing 10% fetal bovine serum (FBS) and antibiotic gentamicin (Gibco, USA) 50 μg/mL at 37 °C in 5% CO_2_ until confluence of 80–90%. The cells from passages 3–5 were used in the experiments. The cells were washed six times with a serum-free DMEM/F12 medium for 20 min each time in a CO_2_ incubator and then cultured for 48 h. Differential centrifugation at 4 °C was used to isolate EVs from conditioned media (CM)^57^. MSC-EVs were isolated from 80 mL of CM to administrate one animal. The same samples were prepared for five rabbits (n = 5). CM was collected and immediately centrifuged at 300×*g* for 10 min to remove floating cells, then at 2000×*g* for 15 min to remove cell debris, and finally at 10,000×*g* for 30 min to remove small debris and huge EVs. After each centrifugation, CM was transferred to a new tube. Then CM was ultracentrifuged at 100,000×*g* for 1.5 h (Optima XPN-100 Ultracentrifuge, Beckman Coulter, California, USA) (Type 70 Ti rotor, Beckman Coulter Optima L-XP) at + 4 °C. Pellet (EVs) was washed with phosphate-buffered saline (PBS) to eliminate contaminating proteins and centrifuged at 100,000×*g* for 1.5 h. Resulting pellet of EVs was resuspended with sterile PBS buffer and snap-frozen at − 80 °C until further use.

### Transmission electron microscopy

To visualize MSC-EVs the transmission electron microscopy (TEM) Libra 120 (Carl Zeiss, Oberkochen, Germany) was performed. To obtain the electron micrographs, the method of negative staining with a 1% aqueous solution of uranyl acetate was used. The sample with MSC-EVs (10 µL) was put on the copper grids coated with formvar/carbon films (Electron Microscopy Sciences, Hatfield, Pennsylvania, USA) for 1.5 min, dried and stained by a 1% aqueous solution of uranyl acetate. In addition, the micrographs obtained by TEM were analyzed using the ImageJ software package for statistical data accumulation (Table S1). To estimate the diameter of MSC-EVs, the area of a circle was determined by ImageJ, and then the diameter was calculated as d = 2*√(S/π) (where S is the area of the circle measured with ImageJ and π is a mathematical constant). The statistical sample for diameter determination included 450 vesicles.

### Determination of MSC-EVs size and protein concentration

The hydrodynamic size of MSC-EVs was assessed by dynamic light scattering (DLS) using a Zetasizer Nano (Malvern Instruments, Malvern, UK). The diameter correction factor was determined by analyzing one size standards (monodisperse polystyrene beads of 60 nm). Total protein of MSC-EVs was evaluated with spectrophotometer Nano Drop One (Fisher Scientific, MA, USA). To determine the total of protein within EVs a Bradford protein assay was applied^58^. Briefly, a standard absorbance curve versus concentration of bovine serum albumin (from 1000 to 10 µg) was prepared. Equal volume of the MSC-EVs sample and Bradford reagent was mixed and incubated 10 min in darkness. Measurement of the absorbance (n = 5) was performed at 595 nm.

### Western blot

Western blot was used to detect some characteristic MSC-EVs proteins. Proteins were separated by electrophoresis using 12% polyacrylamide gels under denaturing conditions in the presence of SDS (SDS-PAGE)^59^. After SDS-PAGE, gels were used for Western blotting^60^. Protein transfers from gel to nitrocellulose membrane (BIO-RAD, 1620112) were carried out in Tris–glycine buffer (pH 8.3) containing 10% ethanol and 0.1% SDS. Following transfer, membranes were washed for 20 min with PBS containing 0.1% Tween 20 and blocked with 5% non-fat dry milk (Biotium, CA, USA) diluted in PBS for 1 h to prevent non-specific binding. Membranes were incubated with primary antibodies at + 4 °C overnight. The primary antibodies used for western blot were anti-Hsp70 antibody (Cat. no. ab181606, Abcam, 1:1000), Anti-CD63 antibody (Cat. no. ab134045, Abcam, 1:1000), anti-CD9 antibody (Cat. no. ab263019, Abcam, 1:1000). The next day, membranes were incubated with HRP-conjugated secondary antibodies (Cell Signaling Technology, 7074P2, 1:10,000) for 1 h at room temperature washed three times on a shaker with PBS containing 0.1% Tween 20, for 5 min each wash. Subsequently, the western blot chemiluminescent signals were enhanced using a SuperSignal West Femto Maximum Sensitivity Substrate kit (34,095, Thermo Scientific, USA) and recorded in signal accumulation mode using a ChemiDoc Touch Imaging System (Bio-Rad, USA).

### Liquid chromatography and mass spectrometry

Home-made trap column (50 × 0.1 mm) was packed with Prontosil 120-C18AQ 5 μm sorbent (Dr. Maisch, Ammerbuch, Germany) and loaded with samples in the loading buffer (2% ACN, 98% H_2_O, 0.1% TFA) at 4 µL/min flow. Samples were separated at RT in a home-packed^42^ fused-silica column (300 × 0.1 mm) filled with Reprosil PUR C18AQ 1.9 (Dr. Maisch, Ammerbuch, Germany) into an emitter prepared with P2000 Laser Puller (Sutter, USA). An Ultimate 3000 Nano LC System (Thermo Fisher Scientific, USA) coupled to the Q Exactive Plus Orbitrap mass spectrometer (Thermo Fisher Scientific, USA) via a nanoelectrospray source (Thermo Fisher Scientific, USA) was used for reverse-phase chromatography. For the elution of peptides, the following solutions were prepared: Buffer A—5% acetonitrile and 0.1% formic acid; buffer B—80% acetonitrile and 0.1% formic acid. Then peptides were loaded in a loading solution (98% 0.1% (v/v) formic acid, 2% (v/v) acetonitrile) and eluted with a linear gradients: 3–35% solution B for 105 min; 35–50% B for 18 min, 50% B during 1 min, 50–99% B for 0.1 min, 99% B during 3 min, 99–2%B for 0.1 min at a flow rate of 500 nl/min. MS1 parameters were as follows: 70 K resolution, 350–1600 scan range, max injection time—35 ms, AGC target—3 × 106. Ions were isolated using a 1.4 m/z window, preferred peptide match and isotope exclusion. The dynamic exclusion time was set to 30 s. MS2 fragmentation was carried out in HCD mode at 17,5 K resolution with HCD collision energy 30%, max injection time—80 ms, AGC target—1 × 105. Other settings: charge exclusion—unassigned, 1, > 7. For subsequent data analysis, raw MS files were analyzed using Peaks studio 10.0 (Bioinformatics Solutions Inc.)^61^. Identification of proteins was made by searching against the Oryctolagus cuniculus Uniprot FASTA database version of 09.07.2021 with a carbamidomethyl Cys as a fixed modification and deamidation Asn/Gln and Met oxidation as variable modifications. False discovery rate for peptide-spectrum matches was determined by searching a reverse database and was set to 0.01. Enzyme specificity was set as C-terminal to arginine and lysine, and a maximum of two missed cleavages were allowed in the database search. Peptide identification was carried out with an allowed initial precursor mass deviation up to 10 ppm and an allowed fragment mass deviation 0.05 Da.

In order to obtain a network of protein–protein interactions (PPI), the list of proteins revealed by MS (Table S2) was loaded into STRING multiple proteins search online tool^62^ with following settings: organism—*Oryctolagus cuniculus*; network type—full STRING network; meaning of network edges—evidence; minimum required interaction score—medium confidence (0.4). As a result, we received a network with the number of nodes equal to 302. Next, for identifying protein complexes the resulting network was clustered using the Markov Cluster Algorithm (MCL) algorithm^63,64^ with an inflation parameter equal to 3. For each cluster with more than two proteins, this name was assigned representing one of three subontologies (biological process, protein complex, or functional group). To graphically display the difference in node degrees, as well as to highlight the interactomes for the previously obtained protein clusters, the PPI network was downloaded from the STRING website and loaded into the Gephi program.

### Animal models

**Figure 6:**
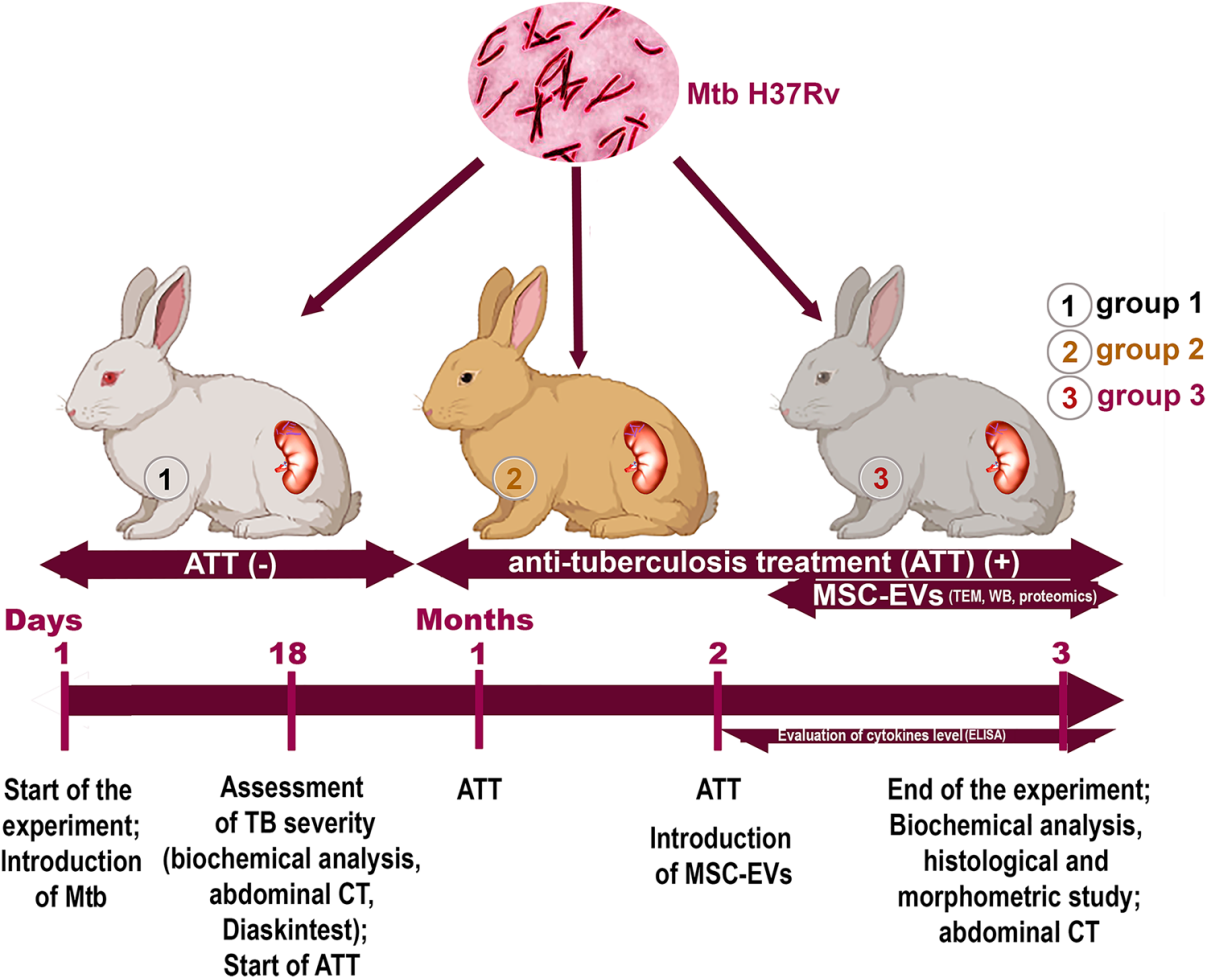
Experimental study design. ATT—anti‐tuberculosis treatment; CT—computed tomography. Examination groups: (1) challenge control (without ATT); (2) standard ATT; (3) standard ATT + MSC-EVs.

The study included the results of a dynamic follow‐up of 15 rabbits (Fig. 6).

The rabbit rTB model was created as described in our previous work^55^. The drug‐susceptible reference strain *Mycobacterium tuberculosis* (*Mtb*) H37Rv at a dose of 10^6^ CFU in 0.2 mL of PBS was injected into the cortical layer of the parenchyma of the left kidney. The development of a specific inflammatory process in the kidneys was confirmed by erythema in response to administration Diaskintest (Generium, Moscow, Russia) in 18 days (data not shown).

Anti-tuberculosis treatment (ATT) was started 18 days after the challenge and continued until the end of the experiment. ATT included oral treatment for 3 months with isoniazid (10 mg/kg, JSC “Moskhimfarmpreparaty named after N.A. Semashko”, Russia); pyrazinamide (15 mg/kg, JSC Pharma sintez, Irkutsk, Russia); ethambutol (20 mg/kg, Shreya Life Sciences, India).

All infected rabbits were divided into three groups (n = 5): the first group is challenge control (without ATT); the second group is standard ATT; the third group is standard ATT + MSC-EVs. Each vial of MSC-EVs contained 0.2 mg/mL of protein in a volume of 0.2 mL was one time injected to rabbit into the lateral vein of the left ear in two months start of the ATT. Each rabbit received one vial with MSC-EVs. All groups of animals were euthanized by an overdose of anesthetic agents injected into the marginal ear vein: sodium thiopental 250 mg (Sintez, Kurgan, Russia) and pipecuronium bromide 1 mg (Veropharm, Moscow, Russia) in 30 days MSC-EVs post-treatment.

### Computed tomography (CT)

To assess the progression of rTB and effectiveness of MSC-EVs treatment CT was performed using a Toshiba One Aquilion tomograph (Toshiba, Japan) on days 18 and 30 after the administration of EVs, respectively. To assess the progression of rTB, healthy rabbit was used as a control. CT was performed by bolus injection of a contrast agent Ultravist‐370 (4 mL at a rate of 1 mL/min) into the marginal ear vein.

### Assessment of rTB severity

Biochemical indicators of blood and changes of body weight were analyzed. Healthy animals (without infection) were used to assess the values of background blood parameters (baseline indicators). Several biochemical indicators of the general inflammatory response and the functional state of the kidneys were analyzed in 18 days after the challenge compared with baseline (healthy animals) and in 3 months after the start of experiment (Table S5). It is known the content of total protein and albumin in the blood depends on the age and intensity of growth of rabbits. The rabbit’s weight during the study was increased compared with the original one. Thus, to get the correct estimate, we divided the values of biochemical parameters such as AL, CR, and CP per gram of weight of the rabbits (except for the activity of ALP, ADA and EL). The activity of purine metabolism enzymes (total activity of adenosine deaminase (ADA), concentration of ceruloplasmin (CP), marker of destruction and remodeling (the activity of serine protease elastase (EL))^65^ and alkaline phosphatase (ALP) were determined. Creatinine (CR) and albumin (AL) concentrations were analyzed with Beckman Coulter reagents on a Synchron CX5 PRO biochemical instrument (Beckman Coulter, USA). In addition, blood samples were carried out at 24 h, 1, 2 and 4 weeks after MSC-EVs administration to evaluate IL-4, IL-10, interferon-gamma (INF-γ) and TNF-α plasma concentration. For each group n = 5. Plasma concentration of IL-4, IL-10, INF-γ and TNF-α were determined with ELISA method according to the manufacturer’s protocol (Cloud-Clone Corp., Texas, USA). Experiment was repeated twice.

### Histology staining

Samples for morphological studies were used after euthanasia by overdose of Zoletil (60 µg/kg) (Virbac, France) and 10 µg/kg Xylazinum (Interchemie werken De Adelaar, B.V., Netherlands) injected into the marginal auricular vein. The kidneys were fixed entirely in 10% formalin solution (pH = 7.0) and cut along the medial axis and a macroscopic description was performed, including the presence/absence of visualized caseous necrotic foci, after which fragments of the middle third of the kidney were excised for further study as the most revealing and including all the studied structures. Sections with a thickness of 1.5–2 μm were made using a rotary microtome HM 325 (Thermo Fisher Scientific, USA). Sections were then deparaffinized, dehydrated, and optionally stained with hematoxylin and eosin and Masson's stain, according to the methods of Van Gieson and Ziehl‐Neelsen. Slides were scanned with a Leica Aperio AT2 scanner (Leica Biosystems, Germany, numerical aperture 0.75) and analyzed using Aperio Image Scope software. Preview and selection of areas for analysis were performed using QuPathv0.2.3 software (University of Edinburgh, UK)^66^. Quantitative morphometry of kidney functional changes was performed on digitized images of histological preparations (Pannoramic MIDI) stained with hematoxylin and eosin or Masson's trichrome using the freely distributed programs Pannoramic Viewer Version 1.15.4 and ORBIT IMAGE ANALYSIS Version 3.64. The information for all cases was provided in Supplementary Fig. S2.

### Statistical analysis

The statistical assessment for evaluated diameter of MSC-EVs, biochemical indicators of plasma by ELISA method, and quantitative indicators of renal functional and structural changes was performed as follows. The values of each indicator were grouped into tables using Microsoft Excel program (Microsoft, USA), the mean (M) and standard deviation (SD) for each group were calculated. For statistical assessment Statistica 7.0 (StatSoft Inc, USA) was used. The significance of the differences was assessed using Mann–Whitney U-criteria. A *p* value < 0.05 was considered to be statistically significant.

### Supplementary Information

Supplementary Information.

## Data Availability

The datasets used and/or analysed during the current study available from the corresponding author on reasonable request.

## Abbreviations

ADA: Adenosine deaminase activity
AL: Albumin
ALP: Alkaline phosphatase
AMPs: Anti-microbial peptides
ANXA1: Annexin A-I
APO A-I: Apolipoprotein A-I
APOE: Apolipoprotein E
ATDs: Anti-tuberculosis drugs
ATT: Anti‐tuberculosis treatment
CP: Ceruloplasmin
CR: Creatinine
EL: Elastase
EVs: Extracellular vesicles
GAL3: Galectin-3
HBB2: Hemoglobin subunit beta-2
HGF: Hepatocyte growth factor
IDO: Indoleamine 2,3-dioxygenase
IL: Interleukin
IGF: Insulin growth factor
KGF: Keratinocytes growth factor
LTF: Lactotransferrin
LZ: Lysozyme
MCL: Markov cluster algorithm
MPs: Macrophages
MSC‐EVs: Mesenchymal stem cell derived extracellular vesicles
*Mtb*: Mycobacterium tuberculosis
NK: Natural killer cells
PGE: Prostaglandin E2
rTB: Renal tuberculosis
TGF: Transforming growth factor
TB: Tuberculosis
TNF-α: Tumor necrosis factor-α
Th2: T helper 2
Thl: T-helper type-l
VEGF: Vascular endothelial growth factor

## References

[1] Bagcchi S (2023). WHO’s global tuberculosis report 2022. Lancet Microbe.

[2] Hutchings MI, Truman AW, Wilkinson B (2019). Antibiotics: Past, present and future. Curr. Opin. Microbiol..

[3] Leise MD, Poterucha JJ, Talwalkar JA (2014). Drug-induced liver injury. Mayo Clin. Proc..

[4] Shamputa IC (2007). Endogenous reactivation and true treatment failure as causes of recurrent tuberculosis in a high incidence setting with a low HIV infection. Trop. Med. Int. Health.

[5] Ramappa V, Aithal GP (2013). Hepatotoxicity related to anti-tuberculosis drugs: Mechanisms and management. J. Clin. Exp. Hepatol..

[6] Kumar R (2010). Antituberculosis therapy-induced acute liver failure: Magnitude, profile, prognosis, and predictors of outcome. Hepatology.

[7] Yan K, Xu G, Li Z (2022). MicroRNA-20b carried by mesenchymal stem cell-derived extracellular vesicles protects alveolar epithelial type II cells from *Mycobacterium tuberculosis* infection in vitro. Infect. Genet. Evol..

[8] Yudintceva N (2022). Mesenchymal stem cells and MSCs-derived extracellular vesicles in infectious diseases: From basic research to clinical practice. Bioengineering (Basel).

[9] Caplan AI, Dennis JE (2006). Mesenchymal stem cells as trophic mediators. J Cell Biochem.

[10] De Jong OG (2014). Extracellular vesicles: Potential roles in regenerative medicine. Front. Immunol..

[11] Elahi FM (2020). Preclinical translation of exosomes derived from mesenchymal stem/stromal cells. Stem Cells.

[12] Börger V (2017). Mesenchymal stem/stromal cell-derived extracellular vesicles and their potential as novel immunomodulatory therapeutic agents. Int. J. Mol. Sci..

[13] Lou G (2017). Mesenchymal stem cell-derived exosomes as a new therapeutic strategy for liver diseases. Exp. Mol. Med..

[14] Murphy DE (2019). Extracellular vesicle-based therapeutics: Natural versus engineered targeting and trafficking. Exp. Mol. Med..

[15] Kooijmans SAA (2016). Modulation of tissue tropism and biological activity of exosomes and other extracellular vesicles: New nanotools for cancer treatment. Pharmacol. Res..

[16] Xu F (2022). Mesenchymal stem cell-derived extracellular vesicles with high PD-L1 expression for autoimmune diseases treatment. Adv. Mater..

[17] Chow L (2020). Antibacterial activity of human mesenchymal stem cells mediated directly by constitutively secreted factors and indirectly by activation of innate immune effector cells. Stem Cells Transl. Med..

[18] Harman RM (2017). Antimicrobial peptides secreted by equine mesenchymal stromal cells inhibit the growth of bacteria commonly found in skin wounds. Stem Cell Res. Ther..

[19] Sutton MT (2016). Antimicrobial properties of mesenchymal stem cells: Therapeutic potential for cystic fibrosis infection, and treatment. Stem Cells Int..

[20] Zhang X (2021). Mesenchymal stem cells and tuberculosis: Clinical challenges and opportunities. Front. Immunol..

[21] Li P, Zhao Y, Ge L (2016). Therapeutic effects of human gingiva-derived mesenchymal stromal cells on murine contact hypersensitivity via prostaglandin E2-EP3 signaling. Stem Cell Res. Ther..

[22] Colombo M, Raposo G, Théry C (2014). Biogenesis, secretion, and intercellular interactions of exosomes and other extracellular vesicles. Annu. Rev. Cell Dev. Biol..

[23] Favaro E (2014). Human mesenchymal stem cell-derived microvesicles modulate T cell response to islet antigen glutamic acid decarboxylase in patients with type 1 diabetes. Diabetologia.

[24] Ren W (2019). Extracellular vesicles secreted by hypoxia pre-challenged mesenchymal stem cells promote non-small cell lung cancer cell growth and mobility as well as macrophage M2 polarization via miR-21-5p delivery. J. Exp. Clin. Cancer Res..

[25] Zhang X (2018). Human gingiva-derived mesenchymal stem cells modulate monocytes/macrophages and alleviate atherosclerosis. Front. Immunol..

[26] Nassar W (2016). Umbilical cord mesenchymal stem cells derived extracellular vesicles can safely ameliorate the progression of chronic kidney diseases. Biomater. Res..

[27] Mokarizadeh A (2012). Microvesicles derived from mesenchymal stem cells: Potent organelles for induction of tolerogenic signaling. Immunol. Lett..

[28] Yudintceva NM (2018). Application of the allogenic mesenchymal stem cells in the therapy of the bladder tuberculosis. J. Tissue Eng. Regen. Med..

[29] Cantaluppi V (2013). Rationale of mesenchymal stem cell therapy in kidney injury. Am. J. Kidney Dis..

[30] Grange C (2019). Stem cell-derived extracellular vesicles inhibit and revert fibrosis progression in a mouse model of diabetic nephropathy. Sci. Rep..

[31] Bruno S (2019). Role of extracellular vesicles in stem cell biology. Am. J. Physiol. Cell Physiol..

[32] Collino F (2015). AKI recovery induced by mesenchymal stromal cell-derived extracellular vesicles carrying MicroRNAs. J. Am. Soc. Nephrol..

[33] Nimiritsky PP (2019). Unveiling mesenchymal stromal cells’ organizing function in regeneration. Int. J. Mol. Sci..

[34] Shi Y (2018). Immunoregulatory mechanisms of mesenchymal stem and stromal cells in inflammatory diseases. Nat. Rev. Nephrol..

[35] Bin A (2018). The ecto-enzymes CD73 and adenosine deaminase modulate 5′-AMP-derived adenosine in myofibroblasts of the rat small intestine. Purinergic Signal.

[36] Bentala H (2002). Removal of phosphate from lipid A as a strategy to detoxify lipopolysaccharide. Shock.

[37] Khundmiri SJ (1997). Effect of reversible and irreversible ischemia on marker enzymes of BBM from renal cortical PT subpopulations. Am. J. Physiol..

[38] Zhu X, Hu J (2022). Adenosine deaminase is a potential molecular marker for diagnosis and prognosis of haemorrhagic fever with renal syndrome. Infect. Drug Resist..

[39] Vallon V, Mühlbauer B, Osswald H (2006). Adenosine and kidney function. Physiol. Rev..

[40] Kim JY (2018). Combined IFN-γ and TNF-α release assay for differentiating active tuberculosis from latent tuberculosis infection. J. Infect..

[41] Roberts VS (2014). The role of adenosine receptors A2A and A2B signaling in renal fibrosis. Kidney Int..

[42] Hochepied T (2003). Alpha(1)-acid glycoprotein: An acute phase protein with inflammatory and immunomodulating properties. Cytokine Growth Factor Rev..

[43] Huang Q (2023). Extracellular vesicle-packaged ILK from mesothelial cells promotes fibroblast activation in peritoneal fibrosis. J. Extracell. Vesicles.

[44] Junttila IS (2018). Tuning the cytokine responses: An update on interleukin (IL)-4 and IL-13 receptor complexes. Front. Immunol..

[45] Labuz D (2021). Interleukin-4 induces the release of opioid peptides from M1 macrophages in pathological pain. J. Neurosci..

[46] Kaufmann SH (2001). How can immunology contribute to the control of tuberculosis?. Nat. Rev. Immunol..

[47] Saraiva M, O'Garra A (2010). The regulation of IL-10 production by immune cells. Nat. Rev. Immunol..

[48] Shaw TC, Thomas LH, Friedland JS (2000). Regulation of IL-10 secretion after phagocytosis of *Mycobacterium tuberculosis* by human monocytic cells. Cytokine.

[49] Geginat J (2016). The light and the dark sides of Interleukin-10 in immune-mediated diseases and cancer. Cytokine Growth Factor Rev..

[50] Hao L (2019). Lactoferrin: Major physiological functions and applications. Curr. Protein Pept. Sci..

[51] Perretti M, Dalli J (2009). Exploiting the Annexin A1 pathway for the development of novel anti-inflammatory therapeutics. Br. J. Pharmacol..

[52] Harrell CR (2019). Mesenchymal stem cell-derived exosomes and other extracellular vesicles as new remedies in the therapy of inflammatory diseases. Cells.

[53] Luan X (2017). Engineering exosomes as refined biological nanoplatforms for drug delivery. Acta Pharmacol. Sin..

[54] Zhou Y (2019). The immunomodulatory functions of mesenchymal stromal/stem cells mediated via paracrine activity. J. Clin. Med..

[55] Muraviov AN (2022). The use of mesenchymal stem cells in the complex treatment of kidney tuberculosis (experimental study). Biomedicines.

[56] Dominici M (2006). Minimal criteria for defining multipotent mesenchymal stromal cells. The international society for cellular therapy position statement. Cytotherapy.

[57] Zhou Y (2019). Injectable extracellular vesicle-released self-assembling peptide nanofiber hydrogel as an enhanced cell-free therapy for tissue regeneration. J. Control Release.

[58] Bradford MM (1976). A rapid and sensitive method for the quantitation of microgram quantities of protein utilizing the principle of protein-dye binding. Anal. Biochem..

[59] Laemmli UK (1970). Cleavage of structural proteins during the assembly of the head of bacteriophage T4. Nature.

[60] Towbin H, Staehelin T, Gordon J (1979). Electrophoretic transfer of proteins from polyacrylamide gels to nitrocellulose sheets: Procedure and some applications. Proc. Natl. Acad. Sci. U. S. A..

[61] Ma B (2003). PEAKS: Powerful software for peptide de novo sequencing by tandem mass spectrometry. Rapid Commun. Mass Spectrom..

[62] Szklarczyk D (2021). The STRING database in 2021: Customizable protein-protein networks, and functional characterization of user-uploaded gene/measurement sets. Nucleic Acids Res..

[63] Spirin V, Mirny LA (2003). Protein complexes and functional modules in molecular networks. Proc. Natl. Acad. Sci. U. S. A..

[64] Wang J (2010). Recent advances in clustering methods for protein interaction networks. BMC Genom..

[65] Visser L, Blout ER (1972). The use of p-nitrophenyl N-tert-butyloxycarbonyl-L-alaninate as substrate for elastase. Biochim. Biophys. Acta.

[66] Bankhead P (2017). QuPath: Open source software for digital pathology image analysis. Sci. Rep..

