# Supplementary material for: Mesenchymal stem cells-derived extracellular vesicles for therapeutics of renal tuberculosis
Source: Sci Rep. 2024 Feb 24;14:4495. doi: 10.1038/s41598-024-54992-z (PMC10894196; doi:10.1038/s41598-024-54992-z)
Supplement: Supplementary file 1 — Supplementary Information. [file 41598_2024_54992_MOESM1_ESM.pdf]

Supplementary information

a

b

c

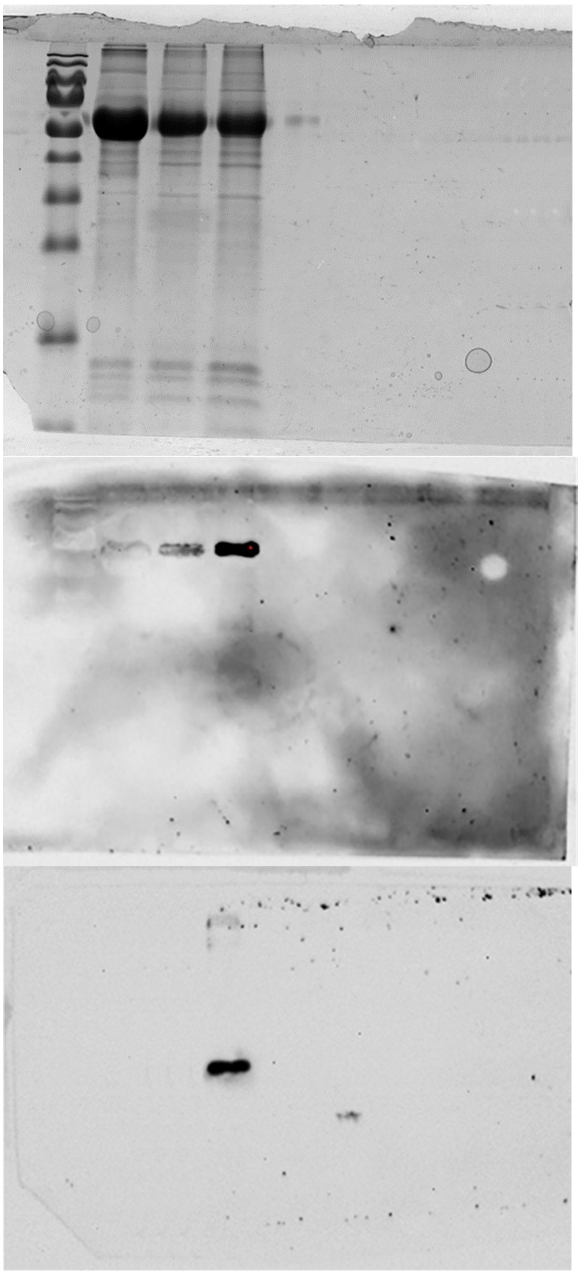

Figure S1. Original raw versions of the SDS-PAGE gel stained with Coomassie Blue **(a)** and the corresponding WB membranes stained with Abs against Hsp70 **(b)** and CD63 **(c)**. The lines from left to right are as follows: the first line is the molecular weight marker (#01161724 (Termo Fisher, USA); Lines 2, 3, 4 – protein samples in loads 30 µg, 35 µg, and 50 µg.

Table S1. EVs proteins revealed by mass spectrometric analysis.

	**Accession**	**-10lgP**	**Coverage (%)**	**Area Sample**	**#Peptides**	**#Unique**	**Avg. Mass**	**Protein name**
1	G1T994	288,28	40	2,70E+08	103	103	338568	Collagen type XII alpha 1 chain
2	G1TN89	282,82	27	7,23E+06	72	9	449577	Heparan sulfate proteoglycan 2
3	A0A5F9CNP8	275,26	26	4,04E+06	65	2	391156	Heparan sulfate proteoglycan 2
4	A0A5F9CJW0	236,97	30	3,63E+07	50	50	257583	Fibronectin
5	A0A5F9C8W6	225,56	33	5,73E+07	32	32	132855	Fibulin 2
6	G1SDN9	217,23	37	2,61E+08	33	33	98591	Prostaglandin F2 receptor inhibitor
7	A0A5F9DU18	207,15	49	1,09E+08	20	20	42874	Milk fat globule EGF and factor V/VIII domain containing

8	A0A5F9DKI8	203,14	55	2,89E+07	18	1	40000	Actin beta
9	G1T2Z5	200,29	26	3,08E+07	24	24	128748	Collagen alpha-2(I) chain
10	G1SL62	198,88	55	6,59E+07	25	25	38776	Annexin
11	A0A5F9CXB0	197,74	27	5,95E+06	25	2	119076	Collagen type I alpha 1 chain
12	A0A5F9C3C4	197,2	31	6,22E+07	24	24	88426	Integrin beta
13	A0A5F9C8F6	196,46	16	2,23E+07	30	30	241237	Tenascin C
14	A0A5F9D7W9	194,85	10	1,19E+08	24	24	265427	Versican
15	G1SWS9	194,79	61	2,35E+07	28	22	53655	Vimentin
16	G1SK79	193,02	19	1,67E+07	19	18	143906	Nidogen 2
17	G1T4A5	192,96	25	7,90E+05	24	1	129191	Collagen type I alpha 1 chain
18	G1T4V7	192,62	13	1,47E+07	35	35	324914	Desmoplakin
19	P62740	188,3	65	2,49E+07	20	10	42009	Actin aortic smooth muscle
20	P50757	185,5	33	2,78E+07	19	19	73803	72 kDa type IV collagenase
21	A0A5F9DBS0	182,42	28	1,43E+07	26	26	113374	Integrin subunit alpha 4
22	G1T9M9	180,4	39	1,06E+07	23	14	70898	Heat shock protein family A (Hsp70) member 8
23	G1SVM1	178,83	28	1,96E+07	22	22	98570	Major vault protein
24	G1SCP8	178,74	41	2,55E+07	28	20	67822	Moesin
25	G1T8D7	178,05	19	1,16E+07	17	17	126546	AE binding protein 1
26	B7NZP9	177,9	12	5,07E+06	23	23	280427	Filamin A alpha isoform 2 (Predicted)
27	G1TAQ7	173,87	21	1,44E+07	17	13	107891	Nidogen 1
28	A0A5F9C7F3	171,13	21	1,78E+07	15	15	92995	Lysyl oxidase homolog
29	A0A5F9DVP3	166,32	14	6,18E+06	22	22	181259	Collagen type XIV alpha 1 chain
30	G1TEG8	166,15	26	9,69E+06	21	21	93815	BRO1 domain-containing protein
31	G1SIJ6	164,73	21	9,53E+06	19	16	108018	Protein tyrosine kinase 7 (inactive)
32	A0A5F9CVI1	163,63	26	7,06E+06	16	16	77094	Annexin
33	G1T2J0	163,59	25	5,78E+06	17	14	81792	Junction plakoglobin
34	U3KPG6	163,38	22	5,05E+07	12	12	57535	Intercellular adhesion molecule 1
35	G1SYJ4	162,33	29	1,08E+07	12	12	47781	phosphopyruvate hydratase
36	U3KNI7	156,43	25	1,45E+07	12	12	64902	5'-nucleotidase
37	A0A5F9DVM4	155,75	18	4,38E+07	10	10	67112	CD44 antigen
38	G1U7K4	152,16	11	1,27E+06	15	6	231504	AHNAK nucleoprotein
39	A0A5F9DES8	150,29	15	6,31E+06	13	13	101154	Laminin subunit gamma 1
40	G1SKF1	147,64	16	4,87E+06	16	15	129767	Thrombospondin 1
41	A0A5F9DJN9	147,55	16	2,84E+06	15	12	110104	Integrin subunit alpha V
42	P11974	146,07	22	4,88E+06	11	11	58048	Pyruvate kinase PKM
43	G1TFU9	145,48	16	4,45E+07	10	10	69257	Galectin-3-binding protein
44	G1TBL6	145,33	9	2,54E+06	12	12	192030	Clathrin heavy chain
45	U3KMU7	143,84	31	6,22E+06	16	11	61814	EH domain containing 1
46	P62160	143,39	52	4,50E+07	10	10	16838	Calmodulin
47	Q9XSC5	142,91	28	1,07E+07	11	11	51851	Clusterin
48	G1SS69	142,88	23	6,30E+06	14	14	85352	C3/C5 convertase
49	G1T1V9	142,76	17	1,75E+05	11	2	69654	Heat shock protein family A (Hsp70) member 2
50	G1TA83	142	39	5,56E+06	13	13	36028	Annexin
51	G1SCI5	141,44	13	3,90E+06	13	13	111571	Myosin IC
52	U3KML1	141,44	27	2,14E+07	9	8	43906	Biglycan
53	G1TED6	141,3	34	1,20E+07	11	11	35946	Annexin
54	A0A5F9DNU2	140,84	16	4,87E+05	12	3	94993	AHNAK nucleoprotein

55	P51662	139,8	29	1,02E+07	12	12	38735	Annexin A1
56	Q28740	139,58	26	1,95E+07	7	7	29078	Basigin
57	G1T2C4	139,49	55	1,53E+07	13	13	22643	Transgelin
58	G1TB50	139,35	43	6,45E+06	7	2	34014	Syndecan binding protein
59	A0A5F9CFU9	139,1	68	1,53E+07	9	9	25332	Brain abundant membrane attached signal protein 1
60	A0A5F9DGE4	139	21	6,56E+06	12	12	80119	Complement C1r
61	A0A5F9DS50	138,74	25	5,74E+06	11	11	72210	Complement C1s
62	G1SXN3	138,48	7	2,58E+06	12	12	191958	Laminin subunit alpha 4
63	D5G340	138,47	59	1,85E+07	11	6	19363	Apolipoprotein E4 (Fragment)
64	G1SJ72	138,44	6	1,89E+06	12	12	246269	Chondroitin sulfate proteoglycan 4
65	G1TJP4	138,32	26	3,34E+06	7	7	42646	Chitinase 3 like 1
66	P46406	138,11	34	9,15E+06	10	10	35780	Glyceraldehyde-3-phosphate dehydrogenase
67	A0A5F9CTS6	137,64	14	3,92E+06	10	7	78442	Nidogen 1
68	P36233	137,58	35	1,08E+07	8	8	31141	SPARC (Fragment)
69	P68105	136,93	22	7,17E+06	9	9	50141	Elongation factor 1-alpha 1
70	P00883	136,2	41	6,98E+06	12	12	39343	Fructose-bisphosphate aldolase A
71	G1TRG8	136,2	30	1,51E+06	9	4	40481	G protein subunit alpha i2
72	A0A5F9C4B7	136,02	33	1,19E+07	10	7	28419	Tyrosine 3-monooxygenase/tryptophan 5-monooxygenase activation protein zeta
73	Q9N0Z6	135,68	10	2,81E+06	9	9	112994	Sodium/potassium-transporting ATPase subunit alpha-1
74	G1T7X6	134,68	9	4,82E+06	15	15	191771	Laminin subunit beta 1
75	G1SL68	134,07	7	1,93E+06	12	12	221678	Myosin heavy chain 9
76	G1SJ77	133,94	54	1,00E+07	10	10	27638	Chloride intracellular channel protein
77	G1TEM7	133,71	38	3,65E+06	12	11	36173	Annexin
78	P15253	133,69	21	3,44E+06	8	8	48275	Calreticulin
79	A0A5F9C1L6	132,75	10	9,98E+05	7	4	96897	Actinin alpha 1
80	G1SCK5	132,72	20	2,58E+06	7	7	46174	Serpin family F member 1
81	G1U729	132,05	15	3,96E+06	9	9	72096	Immunoglobulin superfamily member 8
82	Q9TTC6	131,99	52	1,66E+07	8	8	17837	Peptidyl-prolyl cis-trans isomerase A
83	G1SYI2	131,13	29	1,43E+06	7	4	37377	G protein subunit beta 1
84	G1T4Q9	129,88	23	9,15E+05	5	5	28455	Proteasome subunit beta
85	A0A5F9CXX2	129,56	13	4,18E+06	11	11	113773	Integrin subunit alpha 5
86	G1TCS8	129,3	33	2,82E+06	7	6	22678	RAB1A member RAS oncogene family
87	G1U8B5	128,25	24	2,30E+06	9	9	41289	SRCR domain-containing protein
88	A0A5F9C953	127,93	46	7,08E+06	7	2	26987	Syndecan binding protein
89	Q6GVI2	126,63	39	3,74E+07	6	6	19652	Prepro-alpha-1 collagen type I (Fragment)
90	A0A5F9C848	125,72	21	5,43E+06	7	7	46505	Procollagen C-endopeptidase enhancer
91	G1T8L2	125	8	3,96E+06	9	9	145597	Collagen type V alpha 2 chain
92	P33477	124,19	15	1,97E+06	8	7	54034	Annexin A11
93	G1T567	123,95	32	2,06E+06	6	3	21768	Ras homolog family member A
94	G1TI39	123,94	14	3,26E+05	10	2	71083	Radixin
95	A0A5F9DFE9	123,82	31	7,61E+06	7	7	28877	Ubiquitin specific peptidase 36
96	A0A0G2JH20	123,33	13	5,68E+05	9	4	81365	Heat shock protein 90 alpha family class A member 1
97	A0A5F9D150	122,78	18	4,70E+06	6	6	45571	Fibulin 5
98	G1SR03	122,6	12	2,37E+06	9	9	89322	Transitional endoplasmic reticulum ATPase
99	G1U9D3	122,22	28	2,94E+06	5	5	25498	Vacuolar protein sorting-associated protein 28 homolog
100	G1SHB7	122,18	23	4,75E+06	5	5	32267	ADP-ribosyl cyclase/cyclic ADP-ribose hydrolase

101	G1U7L4	121,97	16	1,74E+06	9	7	71308	78 kDa glucose-regulated protein
102	A0A5F9D4S1	121,83	13	6,81E+05	7	4	73009	Actinin alpha 4
103	P30947	121,36	12	2,97E+05	8	2	83467	Heat shock protein HSP 90-beta
104	G1TKL2	121,3	25	5,43E+06	6	6	23854	EF-hand domain-containing protein
105	G1SQ02	121,16	41	1,11E+07	9	9	22152	Peroxiredoxin-1
106	P49065	120,65	12	2,69E+07	8	8	68910	Albumin
107	Q7YQK3	120,44	23	3,08E+06	11	11	57795	4F2 cell-surface antigen heavy chain
108	G1SP68	120,28	24	6,52E+05	7	2	40546	G protein subunit alpha i3
109	G1TL92	120	11	2,56E+06	6	6	70553	Cadherin 13
110	P18287	118,66	27	1,43E+07	9	4	35497	Apolipoprotein E
111	G1T7R2	118,08	29	7,01E+06	8	7	27433	Tyrosine3-monooxygenase/tryptophan 5-monooxygenase activation protein epsilon
112	P21195	117,83	16	1,72E+06	7	7	56808	Protein disulfide-isomerase
113	G1SH05	117,77	29	7,51E+05	8	2	49727	Tubulin beta chain
114	G1TLE4	117,57	18	1,03E+06	5	2	37331	G protein subunit beta 2
115	G1TQR3	117,34	17	3,28E+06	5	5	46121	Tubulin alpha chain
116	G1SNS5	117,27	22	3,47E+06	8	8	50435	Rab GDP dissociation inhibitor
117	G1SIT9	116,48	37	4,82E+06	9	4	28082	Tyrosine3-monooxygenase/tryptophan 5-monooxygenase activation protein beta
118	G1TA48	115,77	14	4,73E+05	7	4	61096	EH domain containing 4
119	P00939	115,1	39	2,29E+06	9	9	26757	Triosephosphate isomerase
120	Q95215	111,91	14	6,46E+06	10	10	74685	Transforming growth factor-beta-induced protein ig-h3
121	G1TPZ1	111,66	43	7,02E+06	5	5	15008	Galectin
122	G1SFU0	111,53	37	2,28E+06	6	6	23520	RAB7A member RAS oncogene family
123	G1SL07	111,23	39	4,21E+06	7	7	21834	Protein lin-7 homolog
124	G1SER8	110,67	61	2,65E+06	5	5	11297	Profilin
125	A0A5F9C3B7	109,25	9	2,01E+06	9	9	125531	Vinculin
126	G1SGU0	108,97	11	6,18E+05	4	4	56833	CN hydrolase domain-containing protein
127	B7NZQ3	108,76	24	1,24E+06	6	6	34170	Deoxyribonuclease
128	G1T7S0	108,32	30	1,33E+06	5	2	19807	Myosin light chain 12A
129	G1TFT4	107,46	39	1,49E+06	4	4	11407	Vesicle associated membrane protein 3
130	G1T6E8	106,21	17	2,69E+06	6	6	44009	Tumor susceptibility 101
131	Q6DUE5	105,58	27	3,79E+05	4	1	18450	Small GTP-binding protein RhoA (Fragment)
132	G1TZP0	105,23	24	2,38E+06	6	3	28259	Tyrosine3-monooxygenase/tryptophan 5-monooxygenase activation protein gamma
133	G1T0E5	105,1	33	2,10E+06	5	5	23332	Synaptosomal-associated protein
134	G1U4C2	104,96	7	1,64E+06	8	8	103629	Coiled-coil domain containing 80
135	A0A5F9DEF9	104,53	30	2,25E+06	5	5	23474	Glutathione S-transferase
136	G1T3V2	104,3	26	4,81E+06	5	5	22604	Heat shock protein beta-1
137	G1TP66	103,96	15	3,97E+05	9	1	57242	IF rod domain-containing protein
138	A0A5F9DQN3	102,85	7	2,67E+06	6	6	111159	Integrin subunit alpha 3
139	G1SKE7	102,78	5	2,01E+06	6	6	158740	Homeodomain interacting protein kinase 1
140	A0A5F9D7B0	101,93	13	2,33E+06	4	4	48252	Tubulointerstitial nephritis antigen like 1
141	A0A5F9CLR3	101,86	33	2,29E+06	4	4	17223	GLI pathogenesis related 2
142	A0A5F9CC36	101,83	7	6,06E+05	4	4	65755	Endoglin
143	P41975	101,73	21	2,84E+06	4	4	25688	Extracellular superoxide dismutase [Cu-Zn]
144	Q9TV79	101,68	27	1,89E+06	7	7	35961	Tumor necrosis factor receptor superfamily member 6
145	G1SH55	101,57	17	2,11E+06	4	4	29230	Myelin protein zero like 1
146	A0A5F9CQI6	101,57	6	1,08E+06	4	4	105349	Erythrocyte membrane protein band 4.1 like 2

147	G1SZD6	100,89	28	8,78E+05	6	3	27776	14-3-3 protein theta
148	G1T6W4	99,84	21	3,11E+06	7	7	36670	Annexin
149	P47845	99,44	23	4,74E+06	5	5	25502	Galectin-3
150	G1TRY5	98,87	8	8,75E+05	5	5	70839	Plastin 3
151	A0A5F9CQS0	98,63	6	1,42E+05	4	1	78966	Catenin beta 1
152	A5HC35	98,61	18	1,47E+05	4	1	27328	Alpha-v integrin (Fragment)
153	G1SF06	98,48	12	4,37E+05	7	1	60869	EH domain containing 3
154	G1U9R8	97,84	9	1,32E+06	6	6	81396	Gelsolin
155	A0A5F9C4J3	97,23	6	8,19E+05	6	6	125590	VWFA domain-containing protein
156	A0A5F9DC16	96,36	11	1,09E+06	4	1	43322	Protein tyrosine kinase 7 (inactive)
157	A0A5F9CKE3	96,17	23	1,65E+07	5	5	28042	Ferritin
158	G1TEB0	96,1	4	8,15E+05	5	5	128681	Integrin subunit alpha 11
159	G1T6L5	95,01	23	3,29E+06	4	4	22155	SH3 domain binding glutamate rich protein like
160	Q9TT37	93,96	16	2,04E+06	4	4	34940	Sodium/potassium-transporting ATPase subunit beta-1
161	G1SY02	93,88	17	6,66E+05	5	1	49907	Tubulin beta chain
162	B7NZM8	93,6	19	1,21E+06	5	2	28198	Tyrosine3-monooxygenase/tryptophan 5-monooxygenase activation protein eta
163	A0A5F9CWI9	93,28	13	1,74E+05	4	1	37539	G protein subunit beta 4
164	G1U1Q1	93,05	5	1,35E+06	6	5	128205	Thrombospondin 2
165	A0A5F9CB01	92,89	18	2,36E+06	4	4	29056	Neuroplastin
166	A0A5F9D843	92,84	14	1,80E+06	4	4	38577	VPS37C subunit of ESCRT-I
167	G1T6Q0	92,81	2	4,09E+05	3	3	194245	Latent transforming growth factor beta binding protein 2
168	G1SD89	92,72	20	2,14E+06	8	8	47559	VWFA domain-containing protein
169	Q8HY14	92,6	11	3,75E+06	5	5	44247	Poliovirus receptor
170	B7NZD7	92,35	21	2,27E+06	4	4	28657	Proteasome subunit beta
171	A0A5F9CG63	92,3	31	1,25E+06	5	2	15356	RAP1B member of RAS oncogene family
172	G1TMV1	92,11	19	1,60E+06	3	2	18506	Destrin actin depolymerizing factor
173	G1T5H0	92,06	21	8,77E+05	5	5	36468	HtrA serine peptidase 1
174	G1U410	92,03	18	1,39E+05	5	1	49586	Tubulin beta chain
175	G1SDA2	91,68	28	1,81E+06	4	4	15592	Cellular retinoic acid binding protein 1
176	Q9GLC3	90,12	23	1,52E+06	6	6	31593	Sodium/potassium-transporting ATPase subunit beta-3
177	P08628	89,91	30	6,92E+06	3	3	11761	Thioredoxin
178	A0A5F9D229	89,86	6	7,03E+05	4	4	95465	Neuropilin
179	A0A5F9CFE1	89,52	16	1,97E+07	6	6	28545	Tetraspanin
180	A0A5F9D7U7	89,43	15	1,24E+06	3	3	30639	Plasminogen activator urokinase receptor
181	G1SU71	88,84	17	1,98E+06	4	4	26487	Proteasome subunit beta
182	G1TBC1	88,61	6	2,75E+05	4	3	92391	Heat shock protein 90 beta family member 1
183	A0A5F9DHF8	87,41	5	1,14E+06	5	5	106434	Integrin subunit alpha 8
184	G1TRA4	87,09	16	6,87E+05	4	2	21229	NRAS proto-oncogene GTPase
185	P62975	86,99	45	2,90E+07	3	3	8565	Ubiquitin
186	Q28685	86,55	5	1,84E+06	4	4	97030	Dystroglycan 1
187	A0A5F9CGH5	86,47	6	7,41E+05	5	5	94534	Lysyl oxidase homolog
188	G1SD83	86,25	5	6,19E+05	4	4	106531	Integrin subunit alpha 6
189	G1TE69	85,81	7	6,92E+05	3	3	48466	Serine/arginine-rich splicing factor 1
190	A0A5F9CMH5	85,73	36	1,06E+06	4	4	11339	Histone H4
191	G1SVK5	85,61	21	1,46E+07	4	4	15143	Protein S100
192	G1TUD2	85,58	44	2,95E+06	3	3	8006	Guanine nucleotide-binding protein subunit gamma

193	G1SDR2	85,32	23	1,67E+05	4	1	19827	Myosin light chain 9
194	G1SRW4	85,31	6	7,87E+05	5	5	102192	Elastin microfibril interfacer 1
195	G1SV85	85,14	7	1,33E+06	5	5	71554	Monooxygenase DBH like 1
196	A0A5F9CQ70	84,8	27	1,02E+06	5	5	24288	Peroxiredoxin-6
197	P62493	83,99	22	2,54E+06	5	5	24394	Ras-related protein Rab-11A
198	G1U5J7	83,5	11	3,41E+05	7	1	53353	IF rod domain-containing protein
199	G1SVY8	83,32	13	1,28E+06	4	4	44098	creatine kinase
200	Q307R1	82,85	19	5,57E+05	3	3	20310	Peptidyl-prolyl cis-trans isomerase (Fragment)
201	A0A5F9C4V2	82,68	15	2,21E+06	4	4	44566	Cathepsin B
202	Q95MF9	82,22	13	1,39E+06	3	3	26925	Chloride intracellular channel protein 1
203	G1TRZ2	81,98	12	6,97E+06	4	4	44344	Lysosomal associated membrane protein 1
204	G1SZ14	81,47	13	1,57E+06	4	4	36307	Proteasome 20S subunit alpha 3
205	A0A5F9CKA3	81,33	23	8,76E+05	4	2	16917	Cofilin 2
206	G1TUP1	80,85	15	1,03E+06	3	3	21881	Programmed cell death 6
207	Q6X973	80,78	12	4,41E+05	4	2	39515	Guanine nucleotide-binding protein Gs alpha1 (Fragment)
208	A0A5F9DRN8	80,05	15	3,14E+05	4	2	30385	Tropomyosin 2
209	G1T670	79,7	25	1,60E+06	4	4	26395	Proteasome subunit alpha type
210	G1SZ19	79,62	16	1,02E+06	3	2	20745	Ras-related protein Rap-2
211	G1TDB3	78,29	30	1,39E+06	4	4	13611	40S ribosomal protein S25
212	G1SWI7	78,18	10	1,40E+06	3	3	38665	Proteasome 20S subunit alpha 8
213	G1SR48	77,84	4	1,47E+06	4	4	116860	Desmoglein 1
214	G1SNE8	77,73	18	5,87E+06	3	2	21972	Protein S100-A10
215	G1U2E3	77,72	18	6,91E+05	3	3	24980	Zinc finger protein 341
216	U3KM64	77,08	22	1,39E+06	4	4	23687	Clathrin light chain
217	P13491	76,48	12	1,02E+06	4	3	36565	L-lactate dehydrogenase A chain
218	G1T7Z6	76,39	9	4,17E+05	3	3	44633	Phosphoglycerate kinase
219	A0A5F9CC61	76,33	2	6,35E+05	3	3	182022	Collagen type XI alpha 1 chain
220	P20614	75,31	11	5,17E+05	2	2	22937	Metalloproteinase inhibitor 1
221	P16973	75,04	15	2,86E+06	2	2	14722	Lysozyme C
222	Q8WN94	74,91	39	2,58E+06	3	3	9915	Acyl-CoA-binding protein
223	G1T3I9	74,88	10	9,01E+05	5	5	50021	Annexin
224	G1SV22	74,77	20	9,32E+05	3	3	21009	Phosphatidylethanolamine-binding protein 1
225	G1TV79	74,28	11	4,81E+05	2	2	23129	Serpin domain-containing protein
226	A0A5F9C8D3	72,83	19	1,30E+06	3	2	17386	ADF-H domain-containing protein
227	G1TTD6	72,65	12	7,48E+05	3	3	28171	40S ribosomal protein S4
228	G1STA1	72,4	22	1,05E+06	3	3	14757	Golgin A7
229	A0A5F9DDJ9	72,31	20	3,26E+06	5	5	19051	Syndecan
230	P22758	72,1	3	4,40E+05	2	2	91935	Protein-glutamine gamma-glutamyltransferase K
231	Q5U814	71,68	18	1,07E+06	3	2	19948	Cell division control protein 42 homolog (Fragment)
232	Q864T1	71,43	9	2,07E+05	2	2	34526	G-protein beta subunit like-protein (Fragment)
233	G1TFT8	71,17	18	1,02E+06	4	4	23990	Death domain-containing protein
234	A0A5F9CK04	71,12	3	6,07E+05	2	2	95273	N-acetylated alpha-linked acidic dipeptidase 2
235	A0A5F9DLN3	70,82	15	8,99E+05	2	2	16145	ADP ribosylation factor 3
236	A0A5F9CTS1	70,69	14	4,98E+05	2	2	16858	Tetraspanin 14
237	A0A5F9DN13	70,51	25	2,85E+06	2	2	17255	Pleiotrophin
238	G1SI09	70,18	4	4,88E+05	2	2	40436	Calcium homeostasis modulator family member 5

239	A0A5F9CZT5	70,13	16	5,67E+05	3	3	19757	Proteasome subunit alpha type
240	G1TMP1	70,12	6	7,63E+07	4	1	47017	IF rod domain-containing protein
241	A0A5F9DPL1	69,71	20	7,93E+05	6	5	36578	L-lactate dehydrogenase
242	A0A5F9CP75	69,18	20	5,47E+05	3	3	17393	Ribosomal protein S3
243	A5HC45	69,14	11	7,03E+05	3	3	31735	Cathepsin D (Fragment)
244	A0A5F9CIV0	68,79	3	4,91E+05	2	2	73228	Vasorin
245	P62943	67,85	25	1,28E+06	2	2	11951	Peptidyl-prolyl cis-trans isomerase FKBP1A
246	U3KNZ4	67,61	18	1,01E+06	3	3	24592	Nucleoside diphosphate kinase
247	A0A5F9CXB8	67,55	3	2,49E+05	2	2	103890	Calcium/calmodulin dependent serine protein kinase
248	A0A5F9D823	66,38	3	5,27E+05	2	2	75070	Choline transporter-like protein
249	G1SI77	66,2	34	4,75E+05	2	2	11411	S100 calcium binding protein A14
250	G1SHB9	65,95	9	7,17E+05	3	3	43766	Capping actin protein gelsolin like
251	G1TKH3	65,92	20	1,59E+06	4	4	15611	Superoxide dismutase [Cu-Zn]
252	P09451	65,88	17	8,04E+05	2	2	20075	Ferritin light chain
253	G1TAU6	65,52	9	1,12E+06	3	3	40760	Serpin family E member 1
254	U3KME5	65,49	1	1,10E+06	4	4	293422	Fibrillin 1
255	G1TJW1	65,45	17	4,85E+05	3	3	24106	40S ribosomal protein S8
256	A0A5F9DPM1	65,16	7	3,85E+06	4	4	50070	Lysosomal associated membrane protein 2
2572	G1SCY4	64,86	16	1,06E+06	3	3	33007	F-actin-capping protein subunit alpha
258	O19105	64,54	7	4,84E+05	3	3	56659	Neutral amino acid transporter B(0)
259	G1TE13	63,82	16	1,22E+05	2	1	21294	Ras homolog family member G
260	A0A5F9CM11	63,66	11	1,32E+06	3	3	26607	Tetraspanin
261	G1T4X8	63,57	12	9,36E+05	3	3	22823	Proteasome subunit beta
262	B0L419	63,52	10	2,81E+05	2	2	28814	Laminin receptor (Fragment)
263	A0A5F9D5D4	62,89	15	1,04E+06	4	4	34268	Syntaxin 4
264	G1U8S6	62,81	17	8,01E+05	2	2	12753	Vesicle associated membrane protein 5
265	A0A5F9DVE1	62,25	11	3,92E+05	4	2	37229	Tropomyosin 4
266	P24480	61,58	19	4,96E+06	3	2	11429	Protein S100-A11
267	A0A5F9D3T3	61,34	5	7,11E+05	4	4	88676	Discs large MAGUK scaffold protein 1
268	G1STP9	61,14	15	1,49E+06	2	2	18148	Thy-1 cell surface antigen
269	U3KMH6	60,85	4	5,58E+05	3	3	77303	Plakophilin 1
270	A0A5F9DSI6	60,6	3	4,79E+05	4	4	112007	FERM ARH/RhoGEF and pleckstrin domain protein 1
271	A0A5F9DW09	60,04	14	3,91E+05	3	3	23035	Malate dehydrogenase 1
272	G1U1C9	59,95	10	2,15E+06	2	2	34651	Transmembrane protein 119
273	Q01971	59,66	14	3,80E+05	2	2	23532	Ras-related protein Rab-2A
274	G1T673	59,48	16	5,86E+05	3	3	22371	Calcineurin like EF-hand protein 1
275	G1T7Y7	59,48	6	4,97E+05	2	2	33779	F-actin-capping protein subunit beta
276	A0A5F9C3S3	59,16	9	5,72E+05	2	2	35312	dimethylargininase
277	G1U1Q8	59,1	17	2,50E+06	2	2	12359	Macrophage migration inhibitory factor
278	B7NZF1	58,8	5	3,17E+05	2	2	56307	Protein disulfide-isomerase
279	G1TM55	58,6	8	5,76E+05	2	2	28317	40S ribosomal protein S6
280	G1T017	58,56	9	1,08E+06	1	1	24637	Amino acid transporter
281	A0A5F9CT63	58,5	5	2,82E+05	2	2	52011	Angiopoietin like 2
282	G1T159	57,84	15	6,44E+05	3	3	21574	RAS related 2
283	A0A5F9CLG6	57,76	2	7,22E+04	1	1	122847	Neurocan
284	A0A5F9CNX4	57,39	2	1,22E+06	3	3	156557	Alpha-2-macroglobulin
285	G1SRX8	56,73	12	6,53E+05	3	1	48722	Glial fibrillary acidic protein

286	G1SHS7	56,07	4	3,89E+05	3	3	64371	WD repeat domain 1
287	G1TNJ2	56,05	2	4,01E+05	1	1	61022	Zyxin
288	G1TME7	55,66	27	1,53E+06	2	2	8974	Cystatin B
289	A0A5F9DHU0	55,55	3	1,08E+05	1	1	63327	Calnexin
290	A0A5F9D7P4	55,46	3	2,73E+05	2	2	63724	Lamin A/C
291	G1TNF2	55,4	9	5,22E+04	1	1	21270	60S ribosomal protein L17
292	A0A5F9C4F5	55,33	24	3,67E+05	2	2	8731	BRICK1 subunit of SCAR/WAVE actin nucleating complex
293	O77541	54,98	10	3,60E+05	1	1	13870	CD59 glycoprotein
294	G1TYV3	54,31	4	9,58E+04	1	1	48471	Protein kinase C and casein kinase substrate in neurons 3
295	A0A5F9CDD1	53,97	4	8,24E+05	2	2	55285	Prosaposin
296	A0A5F9CVI4	53,83	5	4,18E+05	2	2	41848	Sushi domain-containing protein
297	G1SH76	53,71	15	9,85E+05	2	2	18514	EVA1 domain-containing protein
298	G1TZK6	53,55	5	2,03E+05	1	1	30681	Ig-like domain-containing protein
299	Q0QEN9	53,54	3	1,04E+05	1	1	45578	ATP synthase subunit beta (Fragment)
300	G1SV24	53,5	6	2,05E+05	2	2	37328	Cathepsin V
301	G1SQ03	53,28	2	2,12E+05	1	1	68592	Choline transporter-like protein
302	P01311	53,17	20	7,25E+04	1	1	11838	Insulin
303	A0A5F9CFX8	53,04	2	4,70E+05	2	2	138122	CD109 molecule
304	G1SCD7	52,81	9	2,20E+05	2	2	28197	STI1 domain-containing protein
305	G1SFG7	52,64	1	1,15E+08	2	2	158367	Nestin
306	G1TBW1	52,6	15	4,68E+05	2	2	13928	Thioredoxin domain-containing protein 17
307	A0A5F9DL41	52,42	3	1,16E+05	1	1	72282	EGF-like domain-containing protein
308	G1TVP8	52,15	13	1,10E+06	2	2	13387	Cornifin alpha
309	G1TCW1	51,76	2	2,16E+05	1	1	85622	Transferrin receptor protein 1
310	G1U8L3	51,22	2	2,07E+05	1	1	57221	Alpha-amylase
311	G1TUQ1	50,74	3	1,55E+06	3	3	41276	Syndecan
312	A0A5F9D3N5	50,58	9	6,78E+04	1	1	19021	40S ribosomal protein S24
313	Q9N120	50,48	6	1,49E+05	1	1	34029	Low density lipoprotein-related protein 1 (Fragment)
314	A0A5F9CW48	50,38	4	2,69E+05	2	2	52943	ENAH actin regulator
315	A0A5F9DG45	49,98	6	1,82E+05	1	1	23412	60S ribosomal protein L14
316	A0A5F9C1F4	49,7	2	9,15E+05	2	2	94276	Desmocollin 1
317	P19134	49,63	2	3,81E+05	2	2	76670	Serotransferrin
318	G1TFW8	49,49	3	3,04E+05	2	2	77484	Lactotransferrin
319	G1TMW2	49,37	7	6,01E+05	2	2	30076	Toll interacting protein
320	A0A5F9CIC7	49,29	12	3,67E+05	2	2	17532	EF-hand domain-containing protein
321	G1SVE2	49,28	7	1,28E+05	1	1	19515	Insulin-like growth factor-binding protein 2
322	A0A5F9DB63	49,22	1	6,05E+04	1	1	261276	Talin 1
323	G1SLU0	49,22	5	6,16E+05	1	1	33388	VPS37B subunit of ESCRT-I
324	P30801	49,15	24	1,48E+07	3	3	10154	Protein S100-A6
325	R9W542	49,1	18	8,16E+04	2	2	13903	40S ribosomal protein S18 (Fragment)
326	P54850	49,08	7	1,20E+06	1	1	17758	Epithelial membrane protein 1
327	A0A5F9CZN8	48,89	2	1,52E+05	2	2	217242	Myoferlin
328	A0A5F9DR62	48,68	5	5,03E+05	2	2	36773	IST1 homolog
329	G1T2I5	48,09	3	8,23E+05	3	3	122075	Reticulon
330	A0A5F9DPE8	47,91	8	4,18E+05	2	2	28449	Nucleoplasmin core domain-containing protein
331	G1TXC0	47,89	6	8,69E+05	2	2	27562	Caspase 14

332	G1STH1	47,69	7	4,68E+05	2	2	27098	40S ribosomal protein S3a
333	G1U4H9	47,42	14	2,88E+05	1	1	13735	Tax1-binding protein 3
334	G1TGF1	46,99	8	1,54E+05	1	1	18697	Prostaglandin E synthase 3
335	Q95KM0	46,52	6	1,48E+06	2	2	34449	Arginase-1
336	A0A5F9C5T7	46,37	7	1,15E+05	1	1	18551	S-phase kinase-associated protein 1
337	P02252	46,34	5	3,55E+05	1	1	21897	Histone H1.4
338	Q28709	46,27	7	5,31E+06	2	2	25630	CD63 antigen
339	A0A5F9D586	45,77	4	4,06E+05	2	2	45798	Cell adhesion molecule 1
340	G1SHE6	45,68	18	5,33E+05	2	2	11348	Vesicle associated membrane protein 8
341	A0A5F9CQW8	45,6	17	4,38E+05	2	2	13484	NPC intracellular cholesterol transporter 2
342	G1SL16	45,48	5	2,24E+05	1	1	27412	Serine and arginine rich splicing factor 7
343	Q7YQK4	45,2	3	2,88E+05	1	1	54783	Large neutral amino acids transporter small subunit 1
344	P13280	45,15	6	8,44E+05	2	2	37397	Glycogenin-1
345	Q6Q7K2	45,1	7	4,21E+05	1	1	15398	Midkine
346	A0A5F9CF91	45	8	3,40E+05	1	1	17753	Ribosomal protein L23/L25 N-terminal domain-containing protein
347	A0A5F9C9Q2	44,81	2	2,91E+05	2	2	134925	Collagen type III alpha 1 chain
348	G1SZ91	44,75	10	2,31E+06	2	2	11358	Fatty acid binding protein 5
349	G1SX17	44,73	4	5,70E+05	2	2	47835	Transcobalamin 2
350	G1U155	44,59	21	1,35E+06	3	3	13892	Histone H2B
351	A0A5F9C295	44,5	6	5,14E+05	1	1	22694	Proteasome subunit alpha type
352	A0A5F9CXU7	44,34	15	1,37E+05	1	1	10481	Guanine nucleotide-binding protein subunit gamma
353	A0A5F9D0T9	43,59	2	2,89E+05	2	1	102493	Thrombospondin 4
354	A0A5F9DCP2	43,24	2	3,74E+05	1	1	77076	Transglutaminase 3
355	G1SPD1	42,8	9	2,69E+05	1	1	20274	Myotrophin
356	P01885	42,65	13	4,11E+05	1	1	11654	Beta-2-microglobulin
357	G1TCC1	42,63	3	2,79E+05	1	1	54040	Solute carrier family 16 member 1
358	A0A5F9C2D3	42,55	7	2,64E+05	2	2	83153	Cadherin 11
359	A0A5F9CAX1	42,43	7	5,55E+05	2	2	28225	Biliverdin reductase B
360	O77768	42,28	5	7,79E+04	1	1	33684	Heterogeneous nuclear ribonucleoprotein C
361	P10160	42,1	19	5,45E+05	3	3	16816	Eukaryotic translation initiation factor 5A-1
362	A0A5F9CVY7	41,97	3	5,78E+05	2	2	83969	ADAM metallopeptidase domain 10
363	G1SR53	41,88	2	9,15E+04	1	1	53637	Alpha-L-fucosidase
364	A0A5F9DJP2	41,75	2	2,11E+05	1	1	57289	Catalase
365	Q28888	41,35	5	1,19E+05	2	1	39896	Decorin
366	G1SQM7	40,97	9	3,56E+05	2	2	23707	Activated RNA polymerase II transcriptional coactivator p15
367	G1U1Y5	40,97	9	2,27E+05	1	1	12556	40S ribosomal protein S30
368	P13355	40,96	2	4,14E+05	1	1	54098	Solute carrier family 2 facilitated glucose transporter member 1
369	G1SXP3	40,96	11	2,15E+05	1	1	10451	GST C-terminal domain-containing protein
370	G1SQN7	40,77	2	1,62E+06	1	1	48805	Corneodesmosin
371	A0A5F9D0K2	40,51	4	3,44E+05	2	2	130306	Tripeptidyl-peptidase 2
372	G1TF89	40,5	5	1,18E+05	1	1	20591	60S ribosomal protein L6
373	G1T7R4	40,3	5	7,31E+05	3	3	87994	Integrin beta
374	A0A5F9D4Y6	40,22	7	5,13E+05	2	2	31042	Follistatin like 1
375	G1T432	40,13	2	1,09E+05	1	1	51618	Adenylyl cyclase-associated protein
376	G1SK48	40	3	1,32E+05	1	1	52638	Bleomycin hydrolase

377	G1T1Q1	39,94	3	1,55E+05	1	1	40548	NCCRP1 F-box associated domain containing
378	A0A5F9C9N2	39,59	1	9,14E+04	1	1	101035	Calcium-transporting ATPase
379	G1SFX7	39,52	6	1,02E+05	1	1	20082	ADP-ribosylation factor 6
380	G1TKA4	39,2	11	4,01E+05	1	1	10477	Cysteine-rich and transmembrane domain-containing protein 1
38	G1TXF6	39,16	7	2,08E+05	1	1	15798	60S ribosomal protein L27
382	G1SUV6	38,95	4	1,75E+05	1	1	38734	Neuronal growth regulator 1
383	P79370	38,85	2	5,96E+04	2	1	81214	Rsc protein
384	A0A5F9DGI1	38,5	1	5,43E+05	1	1	103443	Reticulon
385	A0A5F9CYE8	38,29	6	2,19E+05	1	1	18266	Integral membrane protein 2
386	G1TZW4	37,99	7	1,35E+05	1	1	23564	RALA protein
387	G1SST9	37,97	4	1,86E+05	2	1	43281	UV excision repair protein RAD23
388	A0A5F9DUJ9	37,55	2	1,05E+05	1	1	60291	T-complex protein 1 subunit alpha
389	A0A5F9CQG3	37,52	1	1,55E+05	1	1	86993	Solute carrier family 39 member 10
390	A0A5F9DQW2	36,96	5	1,13E+05	1	1	22782	Proteasome subunit beta
391	G1SS79	36,88	8	8,97E+05	2	2	31751	Myristoylated alanine rich protein kinase C substrate
392	G1SZ00	36,58	6	1,63E+05	1	1	25244	Cysteine and glycine rich protein 1
393	A0A5F9DP59	36,16	3	0	1	1	43347	Carboxypeptidase A4
394	A0A5F9D7X3	36,14	4	1,38E+05	1	1	40487	Dynactin subunit 2
395	A0A5F9DFV1	35,74	3	4,00E+05	2	2	82004	Olfactomedin like 2B
396	G1TAC4	35,73	11	4,25E+05	1	1	11954	Glutaredoxin
397	G1T8X6	35,71	3	1,14E+06	1	1	26545	Chromosome 1 open reading frame 68
398	G1TMP9	35,22	9	1,46E+05	1	1	15021	Galectin
399	G1TIT4	35,15	4	3,01E+05	1	1	28473	PSI domain-containing protein
400	G1SNC7	35,11	5	4,14E+05	1	1	24218	Dermatopontin
401	P09809	34,52	4	2,79E+05	1	1	30591	Apolipoprotein A-I
402	P41316	33,9	6	1,28E+05	1	1	20107	Alpha-crystallin B chain
403	A0A5F9DUX5	33,87	12	2,31E+05	1	1	9236	SH3 domain binding glutamate rich protein like 3
404	A0A5F9C4A4	33,78	4	3,03E+05	1	1	34717	phosphopyruvate hydratase
405	P29562	33,54	3	2,18E+05	1	1	45291	Eukaryotic initiation factor 4A-I (Fragment)
406	A0A5F9D2J9	33,29	7	4,56E+05	1	1	15824	Serine and arginine rich splicing factor 5
407	P01948	33,2	5	1,19E+06	1	1	15589	Hemoglobin subunit alpha-1/2
408	G1SI83	33,16	11	2,93E+05	1	1	11202	S100 calcium binding protein A13
409	P68003	33,12	3	1,06E+05	1	1	31581	Voltage-dependent anion-selective channel protein 2
410	A0A5F9DSL3	32,76	2	2,68E+05	1	1	51506	Glycoprotein nmb
411	A0A5F9C5J1	32,7	7	1,83E+05	1	1	14763	Ribosomal protein S5
412	A0A5F9CSP8	32,15	12	4,39E+05	1	1	9939	RAB5A member RAS oncogene family
413	G1SHI6	32,01	8	1,18E+06	1	1	13791	Serum amyloid A protein
414	G1TUX3	31,85	18	1,56E+05	1	1	7613	Rad60/SUMO-like domain-containing protein
415	A0A5F9DKV6	31,77	4	1,62E+05	1	1	26615	Receptor expression-enhancing protein
416	G1SEH1	31,01	3	5,33E+05	1	1	31514	Phospholipid scramblase
417	A0A5F9CFE0	30,98	7	4,26E+05	1	1	15994	Podoplanin
418	G1TB95	30,87	0	1,10E+05	1	1	388048	Sushi von Willebrand factor type A EGF and pentraxin domain containing 1
419	G1TII0	30,62	5	2,26E+05	1	1	23280	Ribosomal protein L15
420	A0A5F9CPL9	30,53	13	8,65E+05	1	1	7318	Guanine nucleotide-binding protein subunit gamma
421	A0A5F9D9B0	29,92	1	2,24E+05	1	1	171745	Fibronectin type III domain containing 1
422	G1SGX4	29,57	5	9,53E+04	1	1	19176	Ribosomal protein S16

423	A0A5F9DDU4	29,1	4	9,28E+04	1	1	60165	Protein 4.1
424	G1ST52	29	2	2,27E+05	1	1	60717	Collagen type XXVIII alpha 1 chain
425	G1SRF7	28,03	1	2,17E+05	1	1	73583	Stress-70 protein mitochondrial
426	G1SK37	27,9	2	8,60E+06	1	1	53170	DNA methyltransferase 1 associated protein 1
427	Q9XSZ4	27,75	1	1,12E+05	1	1	121426	Electrogenic sodium bicarbonate cotransporter 1
428	A0A5F9D7H1	27,69	6	1,75E+05	1	1	18856	GOLD domain-containing protein
429	G1TS06	27,32	8	8,79E+04	1	1	12711	60S ribosomal protein L31
430	G1TVS8	27,13	6	1,32E+05	1	1	15842	Ribosomal protein L13a
431	A0A5F9CWZ2	26,49	2	1,20E+05	1	1	37310	Scavenger receptor class B member 2
432	A0A5F9DI20	26,43	2	1,17E+05	1	1	68184	Adhesion G protein-coupled receptor E5
433	G1TVY5	26,27	1	1,67E+05	1	1	104713	Catenin delta 1
434	A0A5F9CHJ1	26,25	0	1,51E+05	1	1	249223	Notch receptor 2
435	G1TSG1	26,21	9	2,11E+05	1	1	14908	Ribosomal protein L22
436	A0A5F9C4F4	26,2	2	1,43E+05	1	1	55085	UTP--glucose-1-phosphate uridylyltransferase
437	A0A5F9DFV3	25,68	5	1,72E+05	1	1	24217	Ribosomal protein S5 C-terminal domain-containing protein
438	G1TIT6	25,67	7	2,74E+05	1	1	16359	Splicing factor 3b subunit 2
439	A0A5F9DHQ8	25,25	3	3,54E+05	1	1	29250	Leukocyte surface antigen CD47
440	G1SUU8	25,23	2	1,21E+05	1	1	45642	Sushi repeat containing protein X-linked
441	G1U7S4	25,05	4	3,42E+05	1	1	28667	Phosphoglycerate mutase
442	G1SCX2	24,66	5	2,27E+05	1	1	25278	DIRAS family GTPase 2
443	Q28708	24,61	2	1,77E+05	1	1	61482	L-caldesmon
444	A0A5F9DN04	24,54	1	7,35E+04	1	1	95343	N(alpha)-acetyltransferase 15 NatA auxiliary subunit
445	U3KN12	24,45	2	1,17E+06	1	1	42725	Endoplasmic reticulum junction formation protein lunapark
446	G1ST96	24,36	4	1,08E+05	1	1	23946	60S ribosomal protein L13
447	P23035	24,28	2	4,66E+05	1	1	45868	Alpha-1-antiproteinase F
448	A0A5F9D755	24	4	4,35E+05	1	1	18366	Tetraspanin 3
449	G1TP30	23,77	2	3,25E+05	1	1	68024	Ran GTPase activating protein 1
450	P43348	23,64	4	1,29E+05	1	1	19537	Translationally-controlled tumor protein
451	Q8WMQ5	23,57	2	5,35E+05	1	1	55601	Phospholipid transfer protein
452	G1TYL6	23,54	5	1,24E+05	1	1	23428	Ribosomal protein L19
453	A0A5F9C8E1	23,53	3	6,75E+04	1	1	47395	RNA helicase
454	G1SN83	23,52	0	7,35E+04	1	1	196147	Laminin subunit beta 2
455	A0A5F9CZY5	23,22	1	6,54E+04	1	1	156923	Natural killer cell triggering receptor
456	A0A5F9CZS9	23,11	1	6,88E+04	1	1	128100	Collagen type XV alpha 1 chain
457	G1TXF5	23,07	8	2,37E+05	1	1	14332	60S ribosomal protein L35
458	G1SYZ6	22,99	3	1,06E+05	1	1	31242	Transmembrane protein 106B
459	A0A5F9CL61	22,81	4	6,10E+05	1	1	25105	Peptidase S1 domain-containing protein
460	A0A5F9CCH7	22,5	4	1,37E+05	1	1	24893	60S ribosomal protein L7a
461	G1SG07	22,49	1	1,09E+05	1	1	88680	Leucine rich repeat containing 8 VRAC subunit A
462	P06813	22,34	3	1,39E+05	1	1	28239	Calpain small subunit 1
463	P31429	22,29	2	1,25E+05	1	1	45305	Dipeptidase 1
464	B7NZS8	21,83	5	1,60E+05	1	1	22591	40S ribosomal protein S9
465	A0A5F9DDA5	21,76	1	1,07E+05	1	1	104736	Alpha-mannosidase
466	Q5DLV8	21,7	2	2,25E+05	1	1	48916	beta-N-acetylhexosaminidase (Fragment)
467	P02057	21,48	6	1,76E+05	1	1	16133	Hemoglobin subunit beta-1/2
468	A0A5F9C4D4	21,25	1	3,10E+06	1	1	129529	Myosin binding protein C slow type

469	P84246	21,21	5	3,44E+05	1	1	15328	Histone H3.3
470	G1SKT4	21,03	2	1,50E+05	1	1	59754	ATP synthase subunit alpha
471	A0A5F9CAW7	20,6	2	5,63E+05	1	1	48580	Dihydrolipoyllysine-residue succinyltransferase component of 2-oxoglutarate dehydrogenase complex mitochondrial
472	B1PS53	20,17	2	4,84E+05	1	1	46819	Thyroid hormone receptor alpha isoform 1 (Fragment)
473	A0A5F9CWJ5	20,09	3	1,57E+05	1	1	30461	Renin receptor
474	G1TM28	20,07	5	2,51E+05	1	1	16344	UBC core domain-containing protein

Note. Accession, Uniprot accession; -10lgP, MS Score; Coverage (%), Sequence coverage of the protein by identified peptides; Area Sample, The sum of areas of identified unique peptides; #Peptides, The number of identified peptides; #Unique, The number of identified unique peptides.

Table S2. Proteins included in clusters extracted from the MSC-EVs proteome STRING network.

Cluster No.	Cluster members
1	BGN, BST1, CCDC80, COL12A1, COL1A1, COL1A2, COL5A2, COL6A2, COL6A3, CSPG4, DAG1, DCN, DPT, FBN1, HSPG2, IGFBP2, ITGA11, ITGA6, ITGB1, ITGB5, LAMA4, LAMB1, LAMB2, LTBP2, MMP2, NCAN, SERPINE1, SERPINF1, SPARC, SRPX, TGFBI, THBS1, THBS2, TIMP1, ENSOCUP00000013771
2	ALDOA, ANXA4, ANXA5, ATP5A1, CKB, DLST, ENO1, LAMP1, NES, NT5E, PDIA3, PGAM2, PKM, PRDX1, THY1, TPI1, VDAC2, VIM, ENSOCUP00000012659, ENSOCUP00000017094, ENSOCUP00000021667
3	EF1A, EIF4A1, EIF5, HIST1H1E, RPL13A, RPL17, RPL19, RPL22, RPS16, RPS25, RPS6, RPS8, TPT1, ENSOCUP00000006518, ENSOCUP00000006604, ENSOCUP00000015579, ENSOCUP00000016036, ENSOCUP00000019816, ENSOCUP00000021757, LOC100355379, LOC100356974
4	GYG1, H3.3A, PSMA3, PSMA8, PSMB1, PSMB2, PSMB5, RAD23B, TOLLIP, UBC, VCP, LOC100343938, ENSOCUP00000018034, ENSOCUP00000018034, ENSOCUP00000023377
5	ARF6, EHD1, EHD3, EHD4, RAB11A, RAB1A, RAB2A, RAB7A, VAMP8, VAMP3, ENSOCUP00000004655
6	ACTB, CAP1, CSRP1, DSTN, MYH9, MYL12B, PFN1, MYL9, WDR1, ENSOCUP00000010292, LOC100349824
7	AHNAK, ANXA1, ANXA11, ANXA2, ANXA3, ANXA7, ANXA8, S100A11, SVEP1, LGALS1
8	ALB, APOA1, CLU, GSN, CRABP1, SAA3, TRF, INS, ENSOCUP00000015803, ENSOCUP00000025780
9	CRYAB, HSP90AB1, HSP90B1, HSPA2, HSPA8, HSPB1, PTGES3, SUB1, ENSOCUP00000000002
10	CAPNS1, MSN, PTGFRN, RAP2C, RDX, RHOA, RHOG, ZYX, ENSOCUP00000008601
11	CLIC4, GNAI2, GNAI3, GNB1, GNB2, GNG12
12	CDH13, CTNND1, DSP, JUP, PKP1, NCCRP1
13	YWHAB, YWHAE, YWHAG, YWHAH, YWHAQ
14	CAPZA1, CAPZB, DMAP1, MTPN, CLIC1
15	GLRX, HTRA, SOD1, SOD3, TXN
16	SLC16A1, SLC1A4, SLC2A1, SLC3A2, SLC7A5
17	CHMP4B, CHMP4B, TSG101, VPS28, VPS37B
18	BASP, CALM2, MARCKS, RALA
19	TUBB, TUBB2A, TUBB4A, LOC100350967
20	ATP1A1, ATP1B1, ATB1B3, SLC4A4
21	SNAP23, STX4, VAMP5
22	SORT1, TMEM106B, ENSOCUP00000015784
23	FTL, NAALAD2, TFRC
24	HNRNPC, SRSF7, ENSOCUP00000015181
25	HBB2, LTF, Lysozyme
26	ADAM10, IGSF8, SLC44A1
27	BLMH, CASP14, LOC100351764
28	GOLGA7, NRAS, RRAS2
29	CAPG, LGALS3, SSC5D
30	S100A13, S100A14, S100A6
31	CD55, CD59, CFB
32	C1orf68, CDSN, DSG1

Table S3. The statistics of protein clusters extracted from the MSC-EVs proteome STRING network.

Cluster No.	Cluster name	Number of nodes	Number of edges	Average node degree	Average local clustering coefficient	Expected number of edges	PPI enrichment p-value
	Full network	302	2178	14.4	0.462	1187	< 1.0e-16
1	ECM structure	35	244	13.9	0.713	8	< 1.0e-16
2	Energy metabolism	21	77	7.33	0.769	8	< 1.0e-16
3	Ribosomal proteins	21	184	17.5	0.957	35	< 1.0e-16
4	Proteasome complex	14	41	5.86	0.877	9	< 1.0e-14
5	Vesicle transport	11	32	5.82	0.832	2	< 1.0e-16
6	Actomyosin complex	11	28	5.09	0.845	1	< 1.0e-16
7	Annexin group	10	20	4	0.778	0	< 1.0e-16
8	High-density lipoproteins	10	24	4.8	0.802	1	< 1.0e-16
9	Chaperones	9	23	5.11	0.853	1	< 1.0e-16
10	Small GTPases	9	18	4	0.801	2	1.93e-12
11	G protein subunits	6	11	3.67	0.933	1	9.32e-08
12	Adherens junction	6	9	3	0.678	0	2.13e-12
13	14-3-3 family	5	10	4	1	1	5.16e-09
14	Actin capping	5	5	2	0.733	0	3.54e-07
15	Antioxidants	5	6	2.4	0.8	0	2.38e-08
16	Amino acid transport	5	10	4	1	0	< 1.0e-16
17	Endocytosis	4	6	3	1	0	3.01e-11
18	Calmoduline interactors	4	4	2	0.833	0	8.39e-06
19	Tubulines	4	6	3	1	0	3.23e-09
20	Sodium transport	4	5	2.5	0.833	0	4.31e-11
21	SNARE complex	3	3	2	1	0	3.51e-05
22	Sortilin	3	3	1.33	0.667	0	0.000191
23	Ferritin complex	3	2	1.33	0.667	0	0.000171
24	Spliceosome	3	3	2	1	0	0.000465
25	Antimicrobial	3	3	2	1	0	2.24e-07
26	Peptidases	3	3	2	1	0	1.89e-07
27	Proteases	3	2	1.33	0.667	0	7.09e-06
28	Golgi membrane	3	2	1.33	0.667	0	0.0195
29	Galectins	3	2	1.33	0.667	0	9.63e-06
30	S-100 proteins	3	2	1.33	0.667	0	5.01e-07
31	Complement	3	2	1.33	0.667	0	6.46e-06
32	Desmosome	3	3	2	1	0	7.94e-09

Note. Average node degree, a number of how many interactions protein have on the average in the network; Average local clustering coefficient, a measure of how connected are the nodes in the network: highly connected networks have high values; Expected number of edges, how many edges is to be expected if the nodes were to be selected at random; PPI enrichment p-value, a small p-value indicate that the nodes are not random and that the observed number of edges is significant. Extremely low values are shown in exponential notation.

Table S4. Quantitative indicators of the morphological changes of the glomeruli in the left kidney

Indicators	Examination Groups			p
	**1** (n=5)	**2** (n=5)	**3** (n=5)	
Number of glomeruli outside the infection zone. n	11±1.6	9.6±1.9	9.2±1.8	0.22
Parietal sheet of Shumlyansky-Bowman's capsule. µm	3.9±0.7	3.9±0.7	3.9±0.8	0.48
Glomerular capillary diameter. µm	9.8±0.9	5.4±1.1	5.5±1.3	0.92
Area of the glomerulus with the capsule. μm^2	10525±100.9	11922 ±3013	14191±2422	0.22
Area of the renal corpuscle. μm^2	7896±741.9	10332±2098	10430±2082	0.23
Cellularity of the glomerulus. n	52.5±5.11	59.9±4.4	68.4±10.4	0.13

Note: p – significance value for the comparison of second and third groups with the first group

Table S5. Quantitative indicators of the changes of the epithelial cells of the tubules and pelvis of the left kidney.

Indicators	Examination Groups			p
	**1** (n=5)	**2** (n=5)	**3** (n=5)	
Diameter of the proximal convoluted tubule. µm	38.4	42.2±4.2	44.1±3.3	0.46
Diameter of the lumen of the proximal convoluted tubule. µm	10.4	6.7±3.0	10.2±4.8	0.21
Diameter of the distal convoluted tubule. µm	35.6	31.8±2.4	35.5±2.6	0.05
Diameter of the lumen of the distal convoluted tubule. µm	14.5	11.3±4.07	16.9±3.1	0.038
Pelvis wall thickness. µm	743.7	289±198	377±168	0.51
Pelvic epithelium height. µm	74.5	54.5±26.8	66.5±20.1	0.48

Note: p – significance value for the comparison of second and third groups with the first group

Table S6. Quantitative indicators of the vascular changes of the left kidney.

Indicators	Examination Groups			p
	**1** (n=5)	**2** (n=5)	**3** (n=5)	
Interlobular artery diameter. µm	150.6	148.14±43.9	139.9±44.4	0.33
Middle shell thickness of the interlobular artery. µm	15.9	20.4±9.5	17.3±6.7	0.59
Wall thickness of the interlobular artery. µm	48.2	50.66±16.1	41.6±17.9	0.82
Interlobular vein diameter. µm	83.1	114.1±36.3	106.9±46.4	0.28
Wall thickness of the interlobular vein. µm	11.4	6.57±1.86	5.2±2.6	0.96
Peritubular capillary diameter. µm	6.7	8.68±2.46	5.8±1.3	0.23
Wall thickness of the peritubular capillary. µm	1.5	2.55±0.61	2.2±0.60	0.96

Note: p – significance value for the comparison of second and third groups with the first group

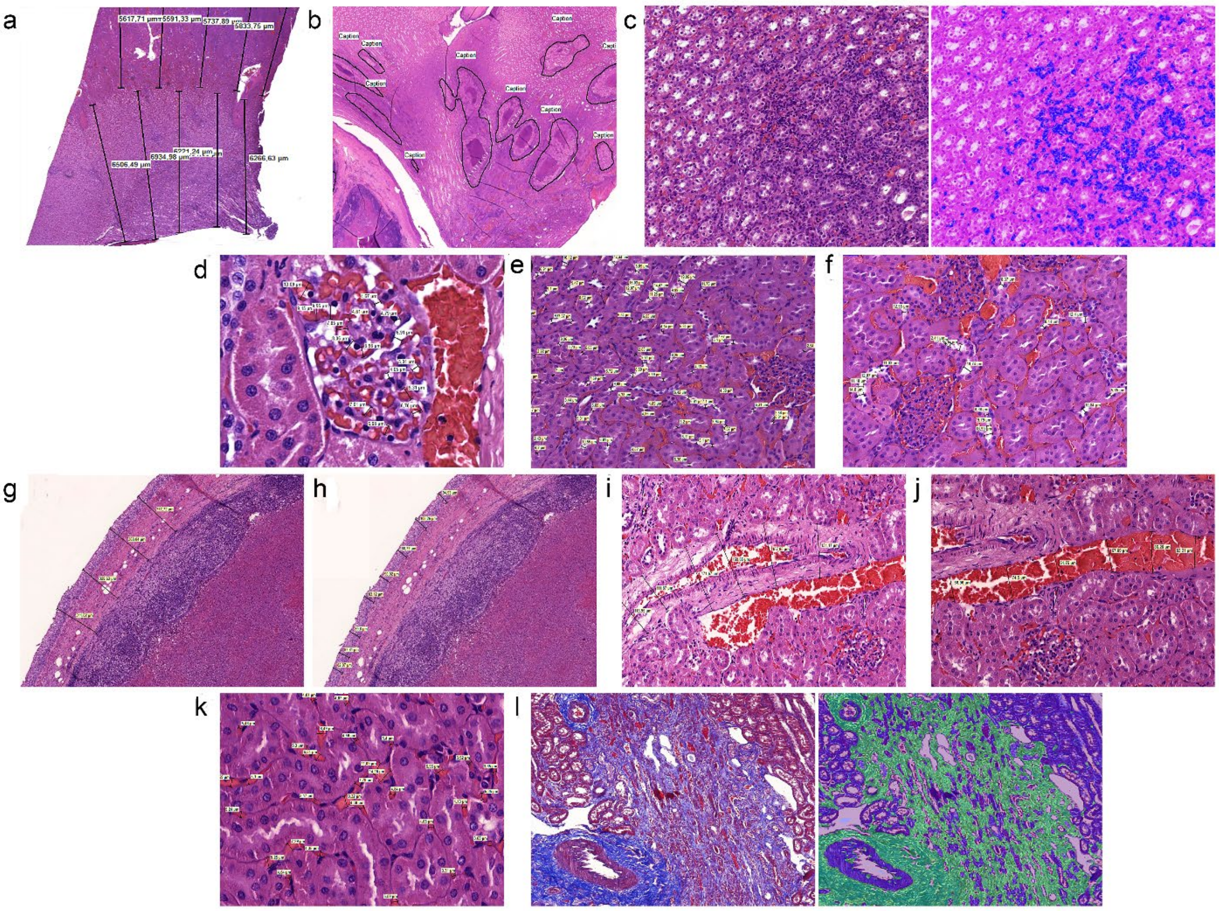

Figure S2. Quantitative morphometry of kidney functional changes. **(a)** Measurements of the thickness of cortical and medulla layers. Assessment of inflammatory infiltration of the kidney cortical and medulla layers. **(b)** The areas of specific and **(c)** interstitial inflammation. The number of glomeruli and nuclei of 10 glomeruli in the region of the renal lobe were counted and the morphometry of the structure of the renal corpuscles, capsules, tubules and capillaries was performed **(d)** to assess glomerular changes. The proximal **(e)** and distal **(f)** lumens of convoluted tubules were measured in a given area of the renal lobe outside the affected area. The thickness of the wall **(g)** and epithelium **(h)** were performed ten times to assess changes in the structure of the renal pelvis. Parameters of the kidney vascular system (diameter and thickness of the wall, middle sheath of interlobular artery **(i)**, interlobular vein **(j)** and peritubular capillaries **(k)** were measured at least ten times. The areas of cortical and medullary stromal collagen were measured in 10 visual fields (five in the cortex and five in the medulla) for each histological slide stained by Masson and expressed as a percentage of the visual field area **(l)**.
